# Cotton plant part 3D segmentation and architectural trait extraction using point voxel convolutional neural networks

**DOI:** 10.1186/s13007-023-00996-1

**Published:** 2023-03-30

**Authors:** Farah Saeed, Shangpeng Sun, Javier Rodriguez-Sanchez, John Snider, Tianming Liu, Changying Li

**Affiliations:** 1 School of Computing, University of Georgia, Athens, Georgia, USA; 2 Department of Bioresource Engineering, McGill University, Montreal, Canada; 3 College of Engineering, University of Georgia, Athens, Georgia, USA; 4 Department of Crop and Soil Science, University of Georgia, Tifton, Georgia, USA; 5 Present Address: Bio-Sensing, Automation & Intelligence Laboratory, Agricultural and Biological Engineering Department, University of Florida, 223 Frazier Rogers Hall, 1741 Museum Road, PO Box 110570, Gainesville, Florida 32611-0570 USA

**Keywords:** Plant architecture, Plant part segmentation, LiDAR, Point cloud, 3D Deep learning

## Abstract

**Background:**

Plant architecture can influence crop yield and quality. Manual extraction of architectural traits is, however, time-consuming, tedious, and error prone. The trait estimation from 3D data addresses occlusion issues with the availability of depth information while deep learning approaches enable learning features without manual design. The goal of this study was to develop a data processing workflow by leveraging 3D deep learning models and a novel 3D data annotation tool to segment cotton plant parts and derive important architectural traits.

**Results:**

The Point Voxel Convolutional Neural Network (PVCNN) combining both point- and voxel-based representations of 3D data shows less time consumption and better segmentation performance than point-based networks. Results indicate that the best mIoU (89.12%) and accuracy (96.19%) with average inference time of 0.88 s were achieved through PVCNN, compared to Pointnet and Pointnet++. On the seven derived architectural traits from segmented parts, an R^2^ value of more than 0.8 and mean absolute percentage error of less than 10% were attained.

**Conclusion:**

This plant part segmentation method based on 3D deep learning enables effective and efficient architectural trait measurement from point clouds, which could be useful to advance plant breeding programs and characterization of in-season developmental traits. The plant part segmentation code is available at https://github.com/UGA-BSAIL/plant_3d_deep_learning.

## Background

Plant architecture is an important factor for enhanced crop yield and quality. It influences light interception, planting patterns, the efficiency of harvest mechanization, and the cost of planting [1]. Cotton plant architecture, for example, affects fiber quality and lint yield [2]. Architectural traits in cotton plants include the number of nodes, the number and location of cotton bolls, and the number of vegetative and fruiting branches, which help to identify genotypic differences and key developmental stages in plant growth [3, 4]. Other architectural traits such as the branch inclination angle, branch length, plant height and internode length may directly impact air circulation and light interception thereby affecting canopy photosynthesis [5]. Therefore, the architectural traits of different crops are studied by plant breeders and geneticists to optimize plant architecture and generate high-yielding varieties [6]. Moreover, plant architectural traits may reveal symptoms of plant diseases and help growers take remedial actions. For instance, significant compression of internodes near the terminal, little-to-no-fruit production, excessive vegetative branching in the bottom of the plant, and abnormal boll size and shape are some symptoms of diseased cotton plants [4]. Although plant architectural traits are important, manual measurements are time-consuming, tedious, and error-prone. Therefore, the ability to measure architectural traits with remote sensing technologies such as automated high-throughput phenotyping is beneficial for crop improvement and management.

To extract plant architectural traits, relevant plant parts need to be segmented from the digital imagery first. Both two-dimensional (2D) and three-dimensional (3D) data have been leveraged to segment plant parts and to derive traits using computer vision techniques in the past. With regard to cotton, one study used 2D RGB images to detect cotton bolls with region-based semantic image segmentation [7]. Later fully convolutional network DeepCotton was designed to perform the same task using deep learning [8]. Another study used ground sensing and performed bloom detection using Faster RCNN [9] and weakly supervised deep learning was used to detect cotton bolls from single plant images [10]. Although aerial imagery methods have been explored extensively in the past, most of them focused on plot level traits estimation, such as yield [11, 12], canopy cover [13], and flowering [13, 14]. Studies based on 2D images have utilized both traditional image processing [15–17] and deep learning-based methods [18–21]. The main disadvantage of the 2D approach is that 2D images can only reveal plant architecture from a single view, leading to challenges such as occlusion and depth ambiguity.

To address these issues, 3D vision offers depth information and includes information from all views, making it possible to accurately estimate plant structural characteristics. Owing to these advantages, researchers have reconstructed 3D data using techniques such as structure-from-motion and shape-from-silhouette methods using RGB imagery [22–24]. Other studies have directly collected 3D data using terrestrial LiDAR scanners [25–28].

Plant part segmentation and trait extraction from 3D data have been studied using traditional point cloud data processing techniques and machine learning methods. Approaches involving region growth and skeleton extraction were used to estimate leaf attributes in sorghum and maize plants [29–33]. In one study, shape fitting and symmetry-based fitting was used to segment branches and leaves to estimate the stem length and leaf area of cotton [34]. Color-based region growth segmentation (CRGS) and voxel cloud connectivity segmentation (VCCS) were used to segment cotton bolls in plot-level data [26]. Various studies used machine learning classifiers such as support vector machine (SVM), K-nearest neighbor (KNN), and Random Forest to segment parts of wheat, barley, sorghum, and tomato plants [35–38]. These methods use handcrafted features (such as fast point feature histogram (FPFH), surface normal, eigenvalues of the covariance matrix) that can successfully distinguish between differently shaped plant parts (such as stems and leaves) in most cases, but these features have not performed well in segmenting similarly shaped plant parts (such as stems and branches both with tubular shapes). In these cases, the identification and utilization of hidden features can significantly improve segmentation performance.

In contrast to traditional machine learning methods, deep learning methods automatically learn features from the data without human design, which can improve the segmentation performance of similarly shaped plant parts. Until recently, the remote sensing and plant phenomics community have begun to investigate 3D deep learning methods for plant part segmentation. For example, a voxel-based deep learning model (3D Unet) was utilized to segment plant parts of the rose bush plant [39]. Other studies used point-based representation for segmentation of wheat, maize, rice panicle and other plant species [40–44]. In other studies, structural traits of rose bush and cucumber plants were extracted by segmenting flower leaves and stems directly from 3D deep learning networks including Pointnet, Pointnet++, DGCNN, and PointCNN [45, 46]. These studies involved dividing the point cloud into blocks and training with them as individual point clouds. Each block was considered as an independent sample while training, but the relation between different blocks was not considered. In some plants, the stems and branches resemble the tubular shape and they are difficult to differentiate in local blocks. Hence, global information of the whole point cloud is useful to distinguish between the main stem and branches. Most of the previous studies have either segmented differently shaped plant parts or showed high time consumption in the segmentation of similarly shaped plant parts (stem and branch). Moreover, they have used either point or voxel data representation. To fill the gaps, this study aims to utilize both point and voxel representations and use point voxel CNN (PVCNN) [47] to achieve efficient segmentation of two similarly shaped parts (main stem and branch) and one differently shaped part (cotton bolls) in an end-to-end manner. The PVCNN leverages point-based representation to perform global feature extraction and voxel-based representation to achieve local feature extraction.

In applying deep learning for plant part segmentation, the annotation of point clouds is an important step to label the data for effective model training. Existing web-based 3D annotation tools such as ‘Semantic segmentation editor’ [48] and ‘Scalabel’ [49] allow pointwise labeling, but the performance is degraded in the case of high-resolution data with millions of points. A lightweight portable software ‘Sustech’ [50] allows loading high-resolution data but it only enables labeling bounding boxes while a pointwise annotation is not possible. Another desktop application ‘Rviz point cloud annotation tool’ [51] leverages the robot operating system (ROS) and allows pointwise annotation using Rviz interface. However, due to intermediate steps, the annotation software based on ROS is less efficient and only limited to Unix-based systems. Therefore, we aim to design a 3D data annotation tool ‘PlantCloud’ to allow efficient annotation and to further optimize the annotation functionality by including both pointwise and bounding box annotations.

The overall goal of this study was to develop a 3D data annotation tool and to perform part segmentation of the main stem, branches, and bolls of cotton plants from point clouds using 3D deep learning models. The specific objectives of this paper were to: (1) design a 3D point cloud data annotation tool PlantCloud for semantic segmentation, (2) segment parts of the cotton plant using both point-based and voxel-based 3D deep learning models and benchmark their performances, (3) develop postprocessing algorithms to correct main stem and branch segmentation errors, and (4) derive seven architectural traits of cotton plants from segmentation results and compare the results with ground truth.

## Results

### 3D annotation software performance evaluation

In contrast to the 3D data annotation tools developed in other studies, PlantCloud software does not require an intermediate desktop application (Table 1). It provides both bounding box annotation and pointwise labeling support whereas other tools cover only one of those features. In terms of user interface features, PlantCloud software consists of property panels and tools for selecting customized label and background color, which is unavailable in other software. Compared to Desktop based Rviz tool, PlantCloud supports both Windows and Unix based systems. In addition, it includes the pan function and point cloud input/output using a file dialog. While web-based tools consist of cross platform support, they require running a web browser and are less efficient. Moreover, the support for displaying RGB values in LiDAR data and file input/output using dialog boxes is only available in one web-based tool.

**Table 1:** Comparison between different 3D annotation tools

Attributes	(a)	(b)	(c)	(d)	(e)
Desktop app		✓			✓
Cross platform support	✓		✓	✓	✓
Bounding box annotation support	✓			✓	✓
Pointwise annotation support		✓	✓		✓
Independent of additional desktop application					✓
Properties panel					✓
File input and save dialog	✓				✓
Background color adjustment					✓
Customized label colors					✓
Pan functionality	✓		✓	✓	✓
Support point cloud rgb values	✓	✓			✓

(a) Sustech [50], (b) Rviz point cloud annotation [51], (c) Semantic segmentation editor [48], (d) Scalabel [49], and (e) our PlantCloud

**Fig. 1:**
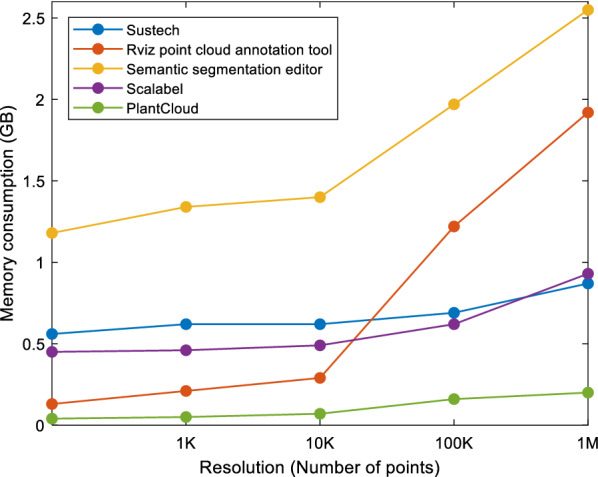
Memory consumption of different annotation tools at different point cloud resolutions

The performance of different annotation software was evaluated by measuring the memory consumption when using the software with point clouds of different resolutions. All software was evaluated on an Ubuntu 20.04 operating system with 16 GB RAM. To estimate the memory consumed by the software for a point cloud of a particular resolution, the RAM usage was recorded before launching the annotation software (stage 1) as well as after launching the software when the point cloud was fully loaded and adjusted (stage 2). We observed that adjusting the point cloud like rotating, translating and zooming impacts memory consumption. We zoomed in on the point cloud so that it was clearly visible. Differences in RAM usage at two stages were considered to be memory consumed by the annotation software. Results indicate that the web-based software consume more memory than the PlantCloud software because of the intermediate web browser application. With the PlantCloud software, memory consumption for high-resolution point cloud with 2 million points was reduced to less than half of the memory consumption by other software (Fig. 1). Specifically, memory consumption by PlantCloud remained less than 250 MB compared to other software that exceeded 800 MB at high-resolution because the PlantCloud software interacts directly with the system’s graphics card using OpenGL specification without an intermediate desktop application. The PlantCloud software also uses less memory than the Rviz point cloud annotation tool. This is because the Rviz annotation tool is based on ROS which requires intermediate execution steps. It is observed that after loading a high-resolution point cloud (> 1 M points) the tools show a decline in performance whereas the PlantCloud software operate smoothly.

### Segmentation results

**Fig. 2:**
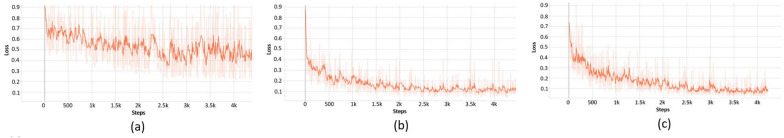
Loss curves for **a** Pointnet, **b** Pointnet++  and **c** PVCNN across training steps. The curve in light orange represents the actual loss values. The curve in bright orange represents moving average of loss values

To assess the performance of the networks, the loss function values during the training of the networks were analyzed. In training, it was observed that the loss function value was highly unstable in case of Pointnet as shown in Fig. 2. Moreover, the fluctuation in loss for PVCNN was higher than the loss for Pointnet++. The moving average of loss was initially higher for PVCNN compared on Pointnet++. Near the end of training, the actual loss value for Pointnet++ dropped below 0.05 whereas the loss for PVCNN dropped even lower to below 0.03. The moving average of loss for PVCNN was around 0.08 while it was around 0.14 for Pointnet++ at the end of the training.

The trained networks were used to segment parts of the cotton plants in the test set, and to analyze and compare overall and class-wise segmentation performance (Table 2). In overall performance, PVCNN achieved the highest mean IoU and accuracy. Both PVCNN and Pointnet++ showed performance superior to Pointnet. PVCNN and Pointnet++ utilize both local and global features of points while Pointnet relies only on global features. Therefore, its mean IoU dropped to less than half of the mean IoU of Pointnet++ and PVCNN. The overall performance of PVCNN was better than Pointnet++  by more than 2% in mean IoU and 0.5% in accuracy. This indicates that PVCNN excels in neighborhood feature aggregation using voxel-based representation compared to Pointnet++ which uses point-based representation.

**Table 2:** Segmentation results from three deep learning networks on the test set

Metric		Pointnet	Pointnet++	PVCNN
Mean IoU (%)		39.68	87.09	**89.12**
Accuracy (%)		70.28	95.57	**96.19**
IoU (%)	Main stem	41.43	84.43	**89.84**
	Branch	7.42	80.42	**81.35**
	Bolls	70.18	**96.44**	96.16
Precision (%)	Main stem	58.16	90.38	**91.82**
	Branch	31.21	91.17	**94.47**
	Bolls	76.27	**98.02**	97.62
Recall (%)	Main stem	64.75	92.24	**97.69**
	Branch	9.24	**87.33**	85.82
	Bolls	90.73	98.32	**98.45**
F1 score (%)	Main stem	61.28	91.3	**94.66**
	Branch	14.26	89.21	**89.94**
	Bolls	82.87	**98.17**	98.03

Bold numbers indicate the best results in the respective category

The class-wise performance of the three trained networks indicates that PVCNN outperformed the other networks in most classes (Table 2). PVCNN showed the highest improvement in the main stem class. It attained a margin of more than 5% IoU and 3% F1 score from the second-best performing network (Pointnet++). While PVCNN achieved the highest IoU and F1 score in both main stem and branch class, the IoU and F1 score of the boll class is slightly lower than that from Pointnet++. Pointnet++ exceeded PVCNN in boll class performance with a small margin of less than 0.5% in both IoU and F1 score. In terms of all classes, Pointnet showed the lowest performance compared to the other networks. Its IoU and F1 score for both the main stem and branch classes was less than half of the other networks. As most of the points in the point cloud belong to the boll class, Pointnet classified most of the branch points as bolls. It showed the lowest IoU for branch class but comparatively higher IoU (70%) for the boll class. Overall, the IoU, precision, recall and F1 score of the boll class from all the networks was higher than that of the main stem and branch classes. This is because both the main stem and branch have similar tube-like shapes whereas cotton bolls have a spherical shape that is distinct from the main stem and branches.

**Fig. 3:**
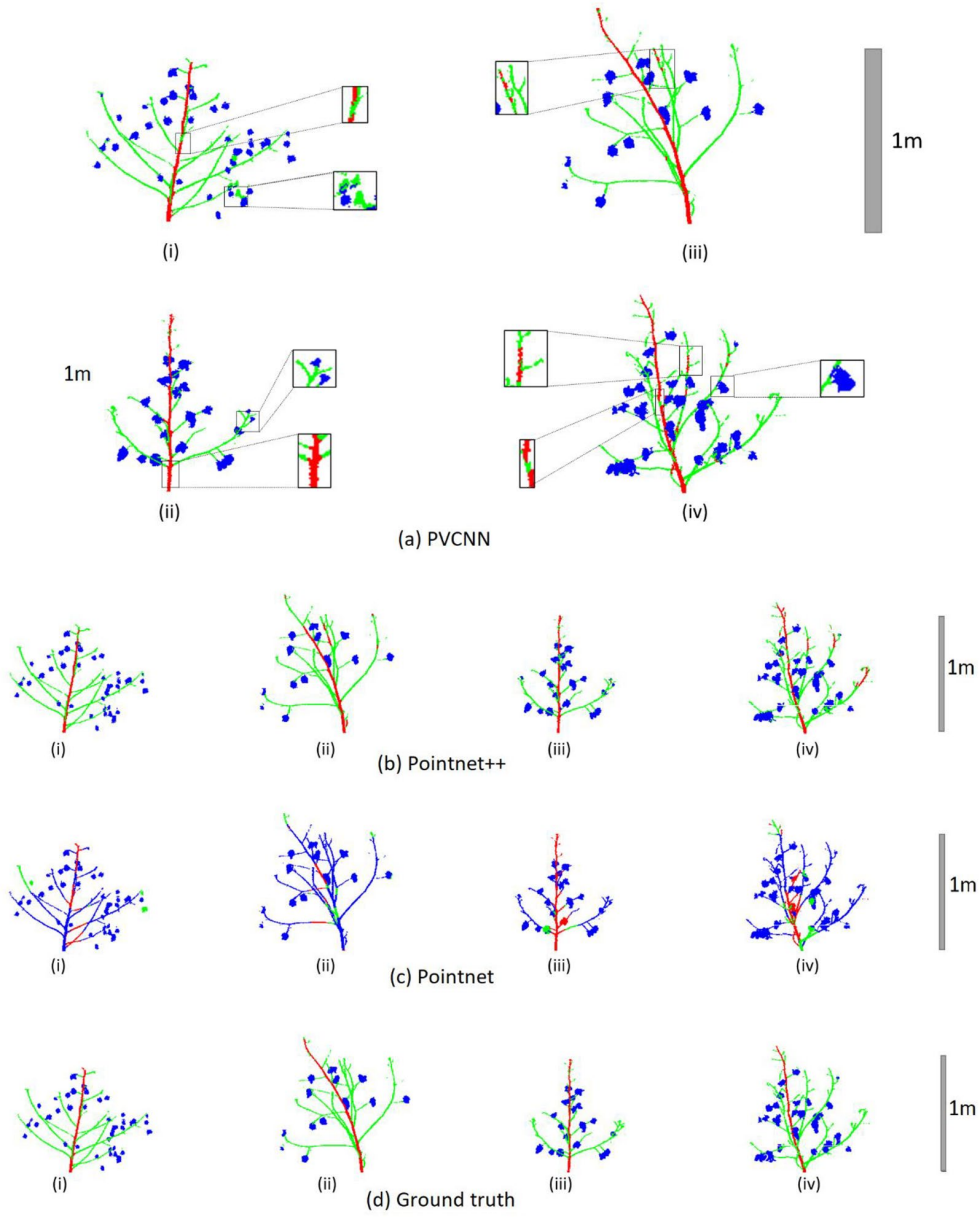
Comparison of segmented point cloud from the three deep learning models with the ground truth. Predicted segments from PVCNN **a**, Pointnet++ **b**, and Pointnet **c** and the ground truth **d** for main stem, branches, cotton bolls in red, green, and blue. Sub-figures (i-iv) represent four representative samples from the test set. Scalebars represent distance in meters

Visualization of inference results show that PVCNN (Fig. 3a) performed better in segmenting main stems, branches, and cotton bolls than Pointnet++ and Pointnet (Fig. 3b, c). While PVCNN and Pointnet++ achieved more than 85% mean IoU, the inference results indicate several mispredictions in both networks. In some cases, PVCNN misclassified part of the main stem as a branch (Fig. 3a-i and a-iv). However, this misprediction is more prominent in the Pointnet++ inference (Fig. 3b-i and b-ii). In other cases, parts of branches were mispredicted as the main stems (Fig. 3a-iii and a-iv). We noticed that these mispredicted branch regions resembled the main stem since they are nearly vertical and show third-order branch attachment. However, this misclassification is less prominent in the PVCNN inference (Fig. 3a-iii and a-iv) than in Pointnet++ inference (Fig. 3b-ii b-iv). Moreover, PVCNN successfully segmented the curved main stem (Fig. 3a-iii) which was not achieved in the other two models (Fig. 3b-ii and c-ii).

Another source of misprediction came from erroneous manual annotation. Because of manual annotation, many peduncles attached to cotton bolls were annotated as bolls and networks learnt to segment them as cotton bolls. As a result, the part of the branch attached to the cotton bolls was mis-segmented in some cases (Fig. 3a-iv). Similarly, networks learnt to classify small floral buds (termed “squares”) at the end of branches as cotton bolls (Fig. 3a-ii). This is because their shape resembles cotton bolls. However, because of their minute size, they are annotated manually as part of the branch in the ground truth.

The inference results of Pointnet (Fig. 3c) validated the quantitative segmentation results in Table 2. As Pointnet had a lower mean IoU, most points were misclassified in Fig. 3c. Among the visualized samples, the main stem was fully segmented in only one plant (Fig. 3c-iii). As Pointnet only utilizes global features, the main stem, branches, and bolls were incorrectly segmented, and most points are classified as boll. The visualization of Pointnet results suggest that cotton bolls were successfully segmented in most cases (Fig. 3c). However, as Pointnet does not include local features it was not able to differentiate between the branch and boll class and most branch parts were mis-segmented as bolls (Fig. 3c-iii).

**Fig. 4:**
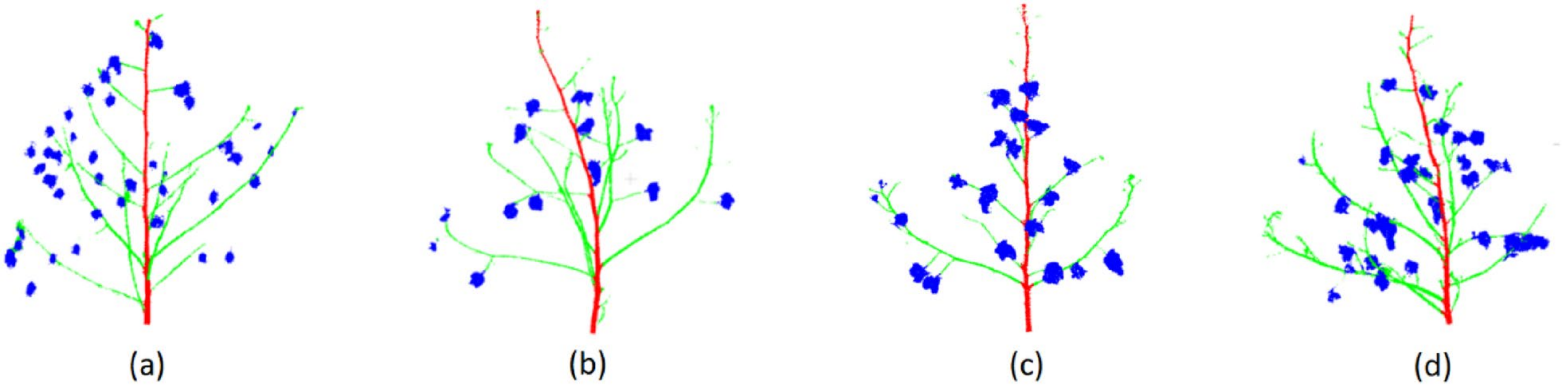
Visualization of PVCNN predictions after postprocessing. The segments for the main stem, branches and bolls are represented in red, green, and blue. Figures (**a**–**d**) represent four random samples from the test set

Further post processing was performed on the segmented results to correct small mis-predicted regions of the main stem and branches. As a result, the branch regions that were mis-predicted as the main stem were corrected (Fig. 4b and d)). This also corrected the small parts in the middle of the main stem that were mis-predicted as branches (Fig. 4a and d).

### Architectural traits extraction results

**Fig. 5:**
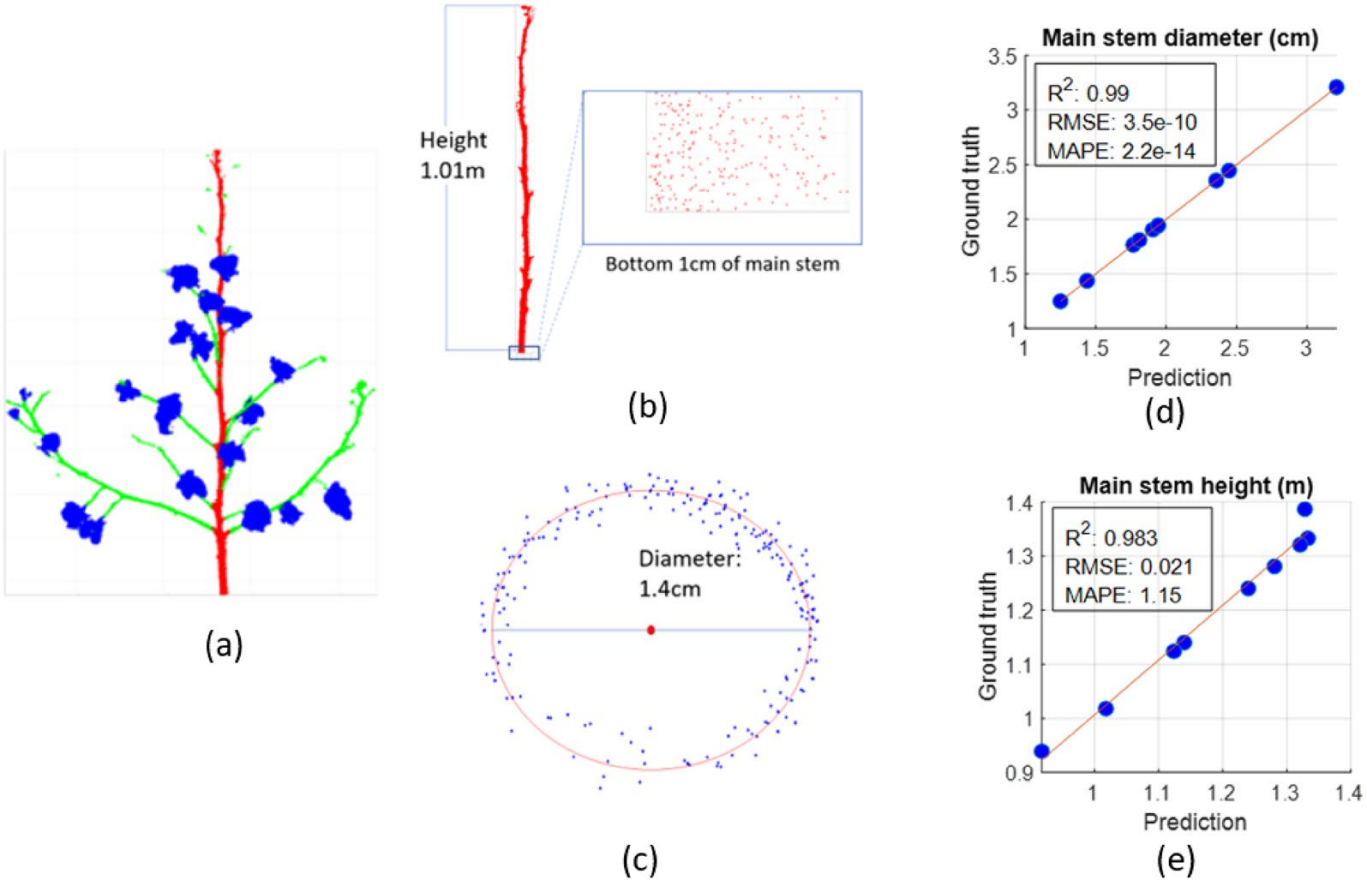
Main stem trait extraction and correlation with the ground truth. **a** Post processed sample from test set. The main stem, branch and bolls are segmented in red, green, and blue, respectively. **b** Main stem height estimation and selection of the bottom 1 cm region. **c** Circle fitting on points projected on the xy plane. **d**, **e** Correlation of main stem diameter and height extracted from predicted and ground truth segments

Given its superior performance, PVCNN was used to segment plant parts from the test set. Afterwards, seven architectural traits were extracted including main stem diameter, main stem height, number of nodes, number of branches, branch inclination angle, branch diameter, and number of bolls. The ground truth values of the traits were extracted from manually annotated segments and compared with the traits extracted from predicted segments. Figure 5a shows the part segments for a sample after applying post processing. We use this example to illustrate the derived architectural traits.

#### Main stem height and diameter

The main stem trait extraction and its procedure in a sample plant are demonstrated in Fig. 5. Because of the accurate prediction of the bottom 1 cm region of main stems in all test cases, the circle fitting was consistent as shown for a sample (Fig. 5b and c). In the overall test set, the main stem traits estimated from the predicted segments showed high correlation with the ground truth with an R^2^ value of more than 0.98 (Fig. 5d and e). In main stem height, low mean absolute percentage error (MAPE) and root mean square error (RMSE) imply that the lowest and highest point in the main stem are accurately classified in most cases. Comparison of the ground truth and predicted values suggest that the main stem height was estimated to be either less than or equal to the ground truth value but did not exceed the ground truth (Fig. 5e). Because the test set covered samples including straight, curved, and tilted main stem, the linear regression results show height extraction of main stems of different orientations with a high correlation (Fig. 5e). As main stem height estimation is based on highest and lowest points, the correct prediction of these points is vital while, other rare mis-segmentations in the middle of the main stem have no effect on the resulting height.

Similar to the main stem height, diameter extraction shows a strong correlation with ground truth (R^2^ = 0.99) and negligible MAPE and RMSE (Fig. 5d). The main stem diameter is estimated using bottom-most 1 cm slice. Therefore, a high correlation of main stem diameters with ground truth values suggests that most points in the bottom region were classified accurately. Moreover, the Pratt method shows consistent results in circle fitting for all plants in the test set. As the Pratt method fits a circle on the main stem predictions in the bottom 1 cm regions of the plant, a few mis-predictions are acceptable because the fitted circle is not affected. However, major mispredictions in the bottom 1 cm regions would lead to an inconsistently fitted circle and inaccurate estimation of the diameter.

#### Number of branches and nodes

**Fig. 6:**
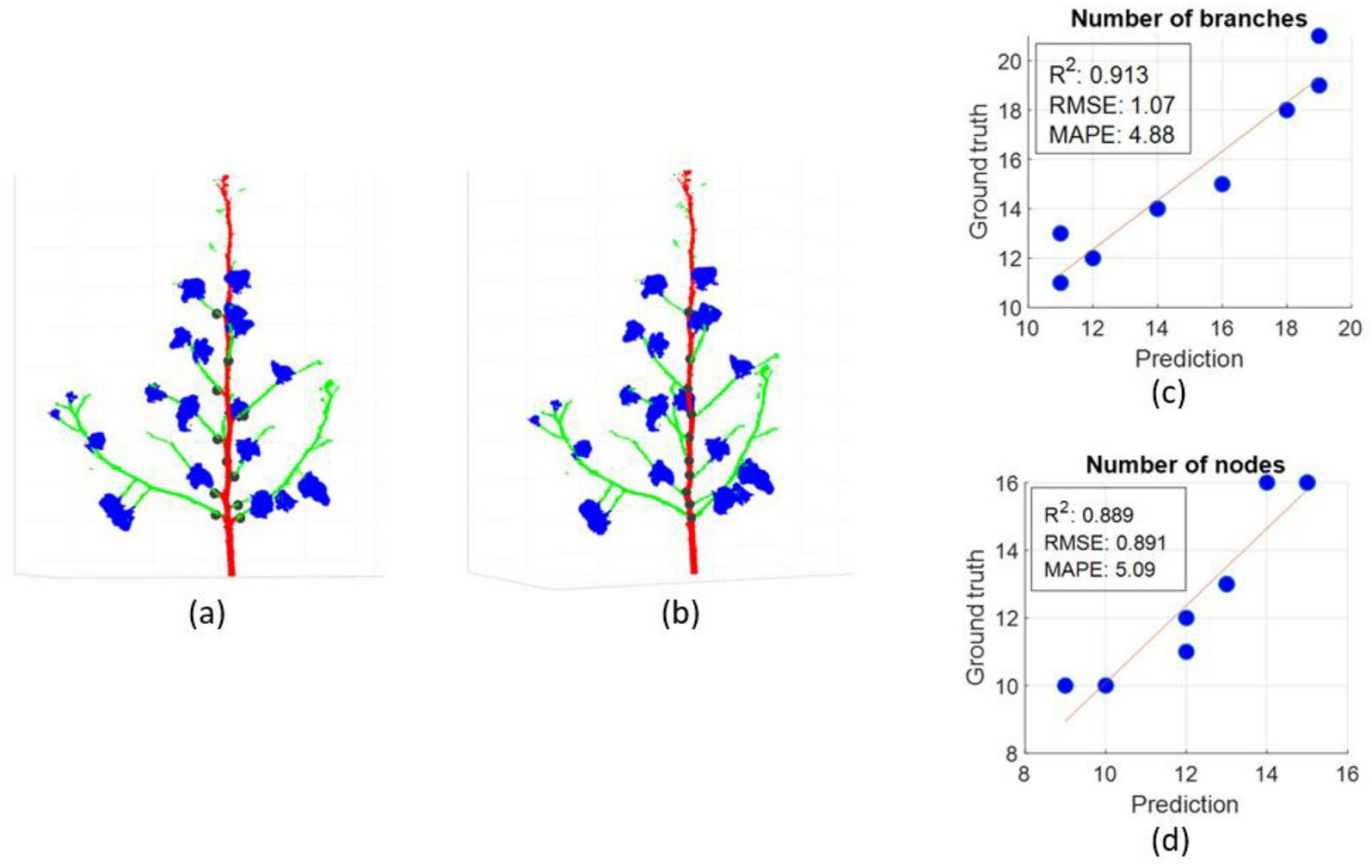
Branch and node trait extraction and correlation with the ground truth. **a** Branch detection. The detected branches shown in black dots. **b** Node detection. The detected nodes are shown in black dots. **c**, **d** Correlation of number of branches and number of nodes extracted from predicted and ground truth segments. Plot represents results for 9 samples (The samples with duplicate ground truth and prediction values are represented as a single data point in the plot)

The detected branches and nodes in the postprocessed plant are illustrated in Fig. 6. Each detected branch was identified by the cluster of green points at branch region adjacent to the main stem and each detected branch location was considered as the average of cluster points within 1 cm from the main stem. Therefore, the circular marks for detected branches do not lie on the main stem but on the branch region adjacent to the main stem (Fig. 6a). For node detection, the circular marks are indicated on the main stem since it was computed by taking an average of points on the main stem slice corresponding to each detected branch cluster. For nearby branches belonging to the same node, the circular mark was computed as the average of the corresponding main stem slices of the nearby branches. As shown in Fig. 6b, the bottom-most two branches belong to the same node, while all other branches each correspond to a distinct node. As the small branches at the top were not considered, the nodes and branches in that region were not detected. The linear regression between the number of branches and nodes estimated from the ground truth and the predicted segments indicates that the branches were correctly detected in most cases (Fig. 6c). The estimated number of branches from the predicted segments differed from the ground truth value by only a couple of branches as indicated by the high correlation (R^2^ = 0.91, RMSE ≈ 1 and MAPE < 5%).

The branch detection results affected the node detection and the estimated number of nodes on a plant. Because of correctly detected branches in most cases, the linear regression between the number of nodes from the ground truth and the predicted segments show a high correlation (R^2^ = 0.88, RMSE < 1 and MAPE < 6%) (Fig. 6d). Similar to the number of branches, the accurate estimation of the number of nodes is highly dependent on the correct prediction of points adjacent to the main stem. As the points far from the main stem do not impact the branch detection, the number of nodes can withstand any misclassification in the region not adjacent to the main stem. Furthermore, the node detection requires only one correctly detected branch at a node and is robust to other missed branches at the same node. As a result, the node detection is not affected by the presence of multiple nearby branches since this was detected as a single node.

#### Branch inclination angle and diameter

**Fig. 7:**
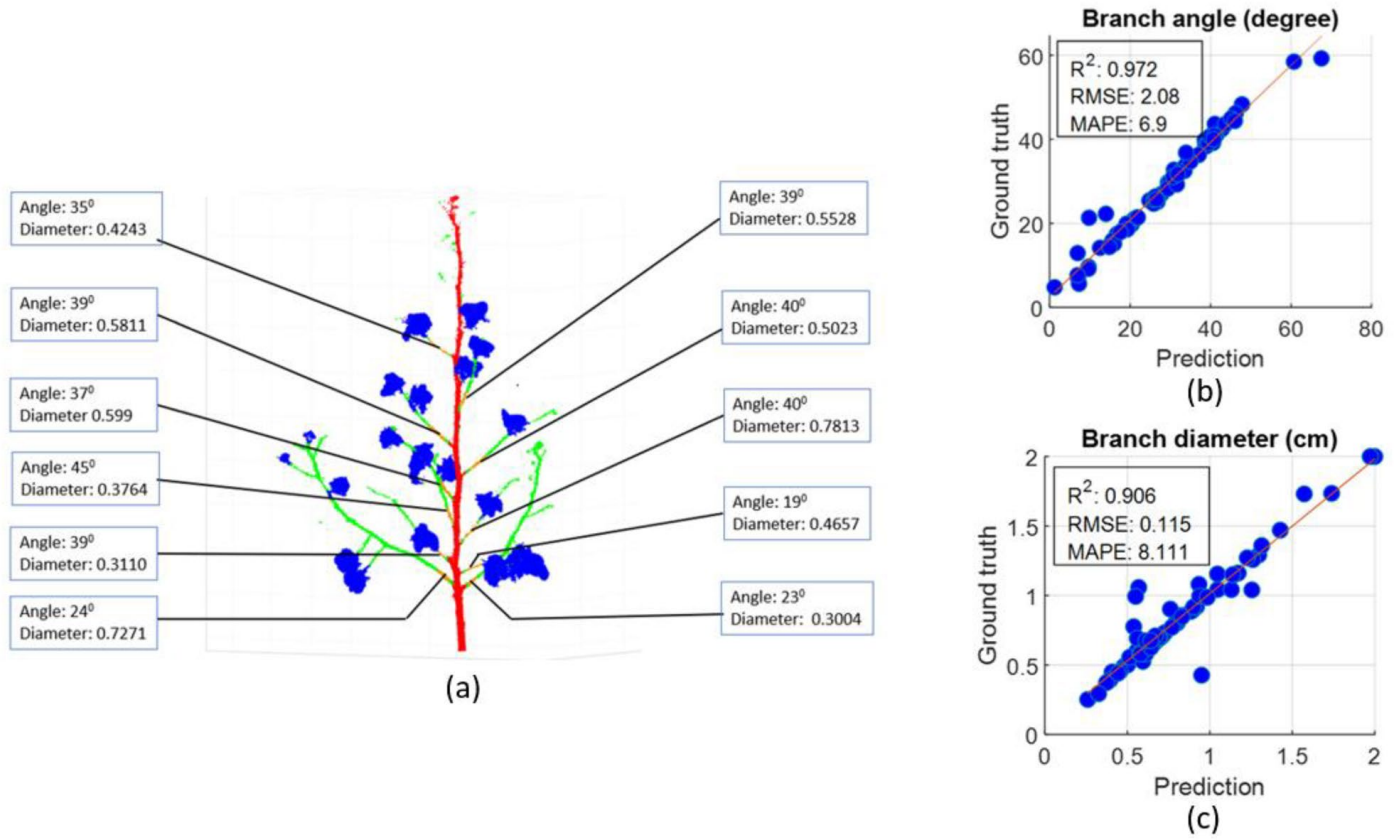
Branch inclination angle (degree) and diameter (cm) estimation, and correlation with the ground truth. **a** represents branch inclination angle and diameter specification for each branch. **b**, **c** represents correlation of branch inclination angle and diameter extracted from ground truth and predicted segments. (Each branch is taken as a separate sample)

The branch inclination angle and branch diameter were estimated for each detected branch in all plants from the testing set. (Fig. 7a). As the bottom most 3 branches were flatter, they typically showed an inclination of less than 25 degrees. On the other hand, the remaining upper branches showed inclination greater than 35 degrees. The branch inclination and diameter estimation were impacted by the branch detection. The linear regression between estimated traits from the ground truth and the predicted segments show a high correlation (R^2^ > 0.9) for branch inclination angle and branch diameter (Fig. 7b and c). The correlation results of the branch diameter between the ground truth and the predicted segments showed higher MAPE (8.1%) than that of branch inclination (6.9%). This is because branch inclination is robust to the small misclassification in the branch attachment region as the principal component indicating the direction of the branch remains almost the same. In contrast, the branch diameter was prone to misclassification in branch attachment region when some branch points are mispredicted as the main stem.

#### Boll number

**Fig. 8:**
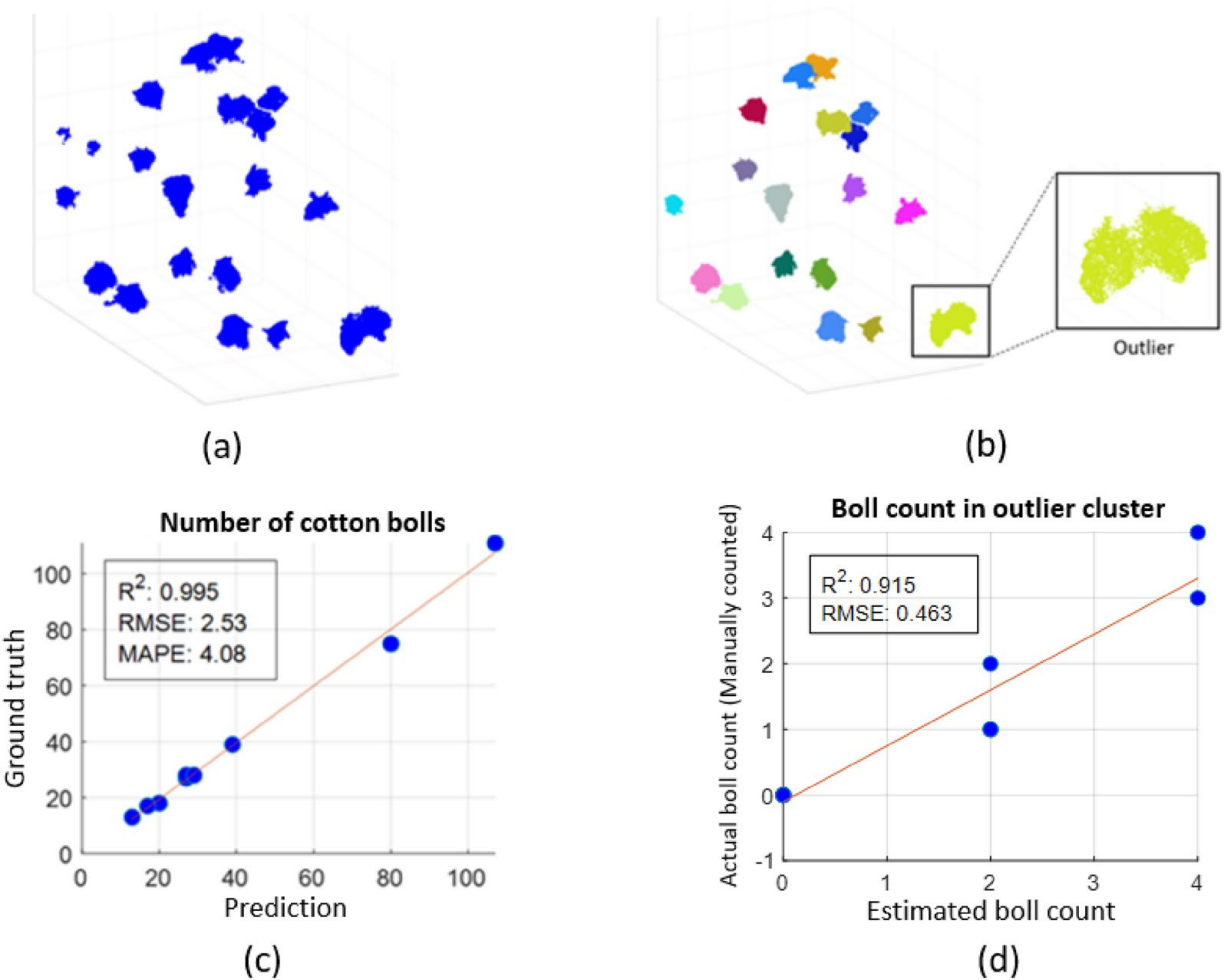
Boll count extraction and correlation with the ground truth. **a** Segmented cotton bolls are represented in blue. **b** Clustering results of cotton boll prediction. Each color represents a cluster. **c** Correlation of number of bolls extracted from ground truth and predicted segments. **d** Correlation of estimated boll count and actual boll count (manually counted) in outlier clusters

One example of boll semantic segmentation from PVCNN and boll counting using clustering is illustrated in Fig. 8. In the DBSCAN results, the clusters having height less than 3 cm are not included (Fig. 8b) which were originally present (Fig. 8a). Each detected cluster corresponds to one cotton boll in most cases (Fig. 8b). However, one outlier cluster representing two connected bolls is detected. The counting for the outlier cluster was performed separately where the size of outlier cluster in number of points was divided by the mean size of all clusters. In most samples, the cotton bolls are far apart and only a few cases of connected bolls exist. Therefore, the outlier clusters were identified successfully in most samples.

The number of cotton bolls shows a high correlation (R^2^ = 0.9) between the ground truth and the predicted segments for applying linear regression (Fig. 8c). Among our samples, three plants with more than 35 cotton bolls were from single-plant plots while the remaining were from regular plant plots (with 10 – 15 plants). In single-plant plots, plants have more space and have more cotton bolls per plant exceeding 100 in some cases, whereas in regular plots, the plants are spaced closely together and produce fewer bolls (< 35 in most cases). As the number of bolls is computed by applying the clustering on boll points, a slight misclassification of branch part near the cotton bolls does not affect the size of clusters significantly and the number of clusters remains the same.

We observed that our algorithm is robust to most outlier clusters and it correctly estimates the boll counts in them. To analyze the sensitivity towards the outlier clusters, we selected all outlier clusters from the test samples and compared the estimated and actual (manually counted) boll count value for them. Linear regression applied to explore the correlation between the two values shows a high R2 value of more than 0.9 and RMSE of less than 0.5 as shown in Fig. 8d.

## Discussion

In terms of data collection, we ensured several conditions to achieve high-quality LiDAR data. Firstly, LiDAR scanning was performed on single plants without nearby plants so that the scan was not affected by occlusion from neighboring plants and shadowing. Moreover, the scan quality could be affected by the wind which causes the same parts to be recorded at slightly different locations. To mitigate the wind effect, the data were collected in calm weather for samples scanned in an outdoor environment. To address the uneven and uncontrolled illumination conditions, only coordinate values excluding the RGB values were utilized in the method. As the movement causes vibrations and affects the quality of collected scans, we set the instrument in a stationary position in both indoor and outdoor environments to achieve the highest data quality. As the FARO LiDAR sensor is limited in its ability to capture very thin parts, it showed missing data in the case of rarely occurring very thin branches. We observed that all cotton bolls due to their spherical shape were captured including smaller bolls from single-plant plots and bigger bolls from plants selected from multi-plant plots. Due to defoliated plants, we observed that cotton bolls had very little to no occlusion in point cloud samples. The plants were scanned from multiple overlapping views so that part of the cotton boll occluded from one view may be captured in another view. The FARO LiDAR scanner device was able to preserve the scale of the object when placed at different distances from the plant. In this study, we measured the distance among the points in the configured measurement unit which was set to ‘meters’. For this study, annotated and preprocessed point clouds along with raw scans were generated and the digitization footprint [52] is around 5.1 gigabytes.

**Fig. 9:**
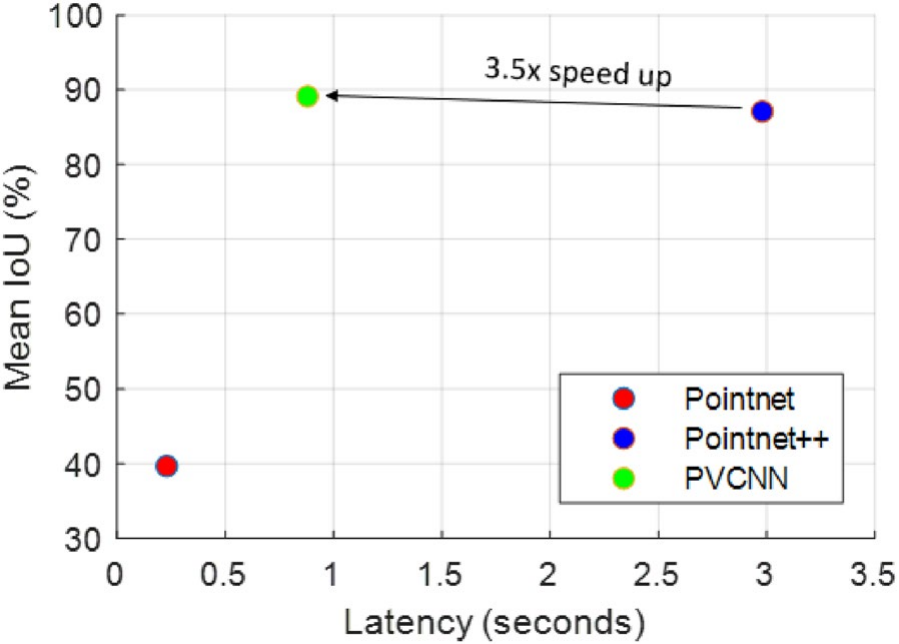
Mean IoU vs measured latency of three 3D deep learning models

We have demonstrated that PVCNN achieved optimal performance in terms of inference accuracy and efficiency: with an mIoU of more than 89% while consuming the least amount of time (less than 1 s) (Fig. 9). The point-based 3D deep learning models took permutation invariant point clouds as input, which occupied less memory but take more time to compute since data are not saved in adjacent memory locations. Pointnet++ consumed the most time because it applies multiple set abstraction layers in a sequence and aggregates neighborhood information from point-based representation. In comparison, voxel-based 3D models take grid representation (i.e., pixels in 3D) as input and 2D convolution operations can be readily extended to 3D and applied on the voxels. PVCNN not only utilizes point-based representation to extract global features, but also uses voxel-based representation for neighborhood aggregation in which data are organized in memory; therefore the PVCNN is less time-consuming with a speed up of 3.5 times from Pointnet++. However, Pointnet showed the least time consumption but had the lowest mIoU because of the absence of local feature extraction.

3D deep neural network architecture plays a critical role in the segmentation results. In our approach, various network architectures for Pointnet++ and PVCNN were manually tried and tested to observe the segmentation results and the neural network architecture that showed the best results in our experiments was finalized. However, the task of finding the best-performing architecture is an optimization problem that can be achieved through a neural architecture search, and it is planned for future research to further improve the results. The data set used in this study consists of point clouds from 30 plants which are annotated manually. The performance of transfer learning was observed by first training the network on the Shapenet dataset and using the learned weights to perform further training on our plant dataset. However, because of the difference in the scale of parts in the two datasets, transfer learning reduced the overall performance. The plant dataset includes a variety of plant architectures from different genotypes including curved, tilted and straight main stems. To introduce more variety in the dataset, data augmentation was applied in every training iteration to rotate the plants along the x and y axes. We notice that data augmentation achieved an improvement of around 1% mIOU and the network could identify all straight, curved and tilted main stem types. Due to the time-consuming and labor-intensive task of data collection and point-wise annotation of point clouds, the size of the current data set is limited. The expansion of dataset leveraging geometrical shapes for fake data generation and for labeled plant parts is another research area which may further improve the segmentation results.

In terms of all extracted architecture traits, this study demonstrates a mean absolute percentage error of less than 10%. The architecture traits were computed for plants from both single and multi-plant plots. The boll number was estimated with the highest R^2^ value (0.995) and shows potential to be utilized by cotton growers and breeders in plant physiology studies. The procedure correctly detected the disjointed (far apart) bolls in most cases but misclassified small floral buds (termed “squares”) as bolls. Because of this, our method removes all detections with a height of less than 3 cm including both very small cotton bolls and squares. To differentiate between very small cotton bolls and squares, the RGB information can be leveraged. Our current method utilized only the x, y, and z coordinates without RGB information. Therefore, it is invariant to illumination conditions and can be performed in both indoor and outdoor settings.

**Fig. 10:**
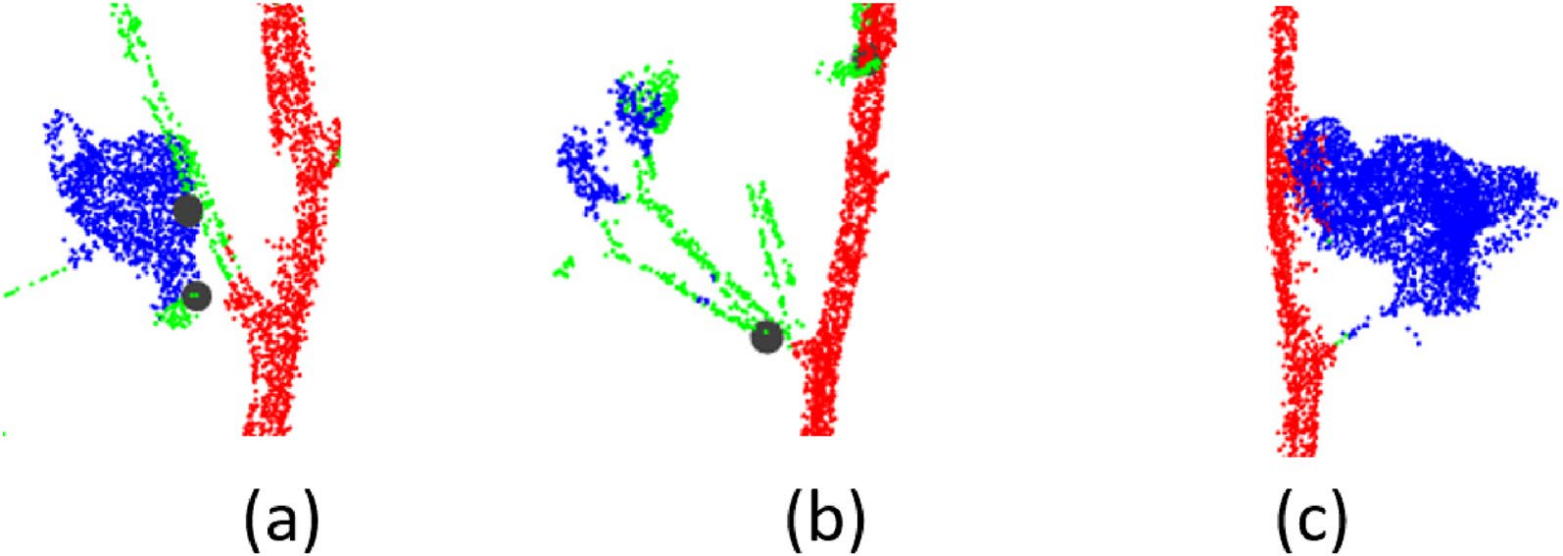
A few erroneous cases in branch detection. Circular marks represent detected branches. **a** Incorrectly detected branch (bottom circular mark) due to mispredicted boll region. **b** Multiple nearby branches detected as a single branch. **c** Missed branch detection due to mispredicted branch region

Architectural traits related to the branches (including the number of nodes, branch diameter, branch angle, and branch count) were estimated with high accuracy because of correctly detected branches in most cases. The few cases of missed and incorrectly detected branches resulted in small errors in branch-related traits (Fig. 10). Incorrect branches were detected in cases where the method incorrectly segmented the branch region near the main stem (Fig. 10a). In some scenarios, the method detected multiple nearby branches at the attachment point as a single branch and resulted in underestimation of the branch and the node count (Fig. 10b). In other cases the method did not correctly identify the branch points near the main stem and failed to detect the branch (Fig. 10c). Overall, due to the low error in the detected branches, the method showed a high correlation (R^2^ > 0.8) between the ground truth and the predicted values of the branch related traits.

**Fig. 11:**
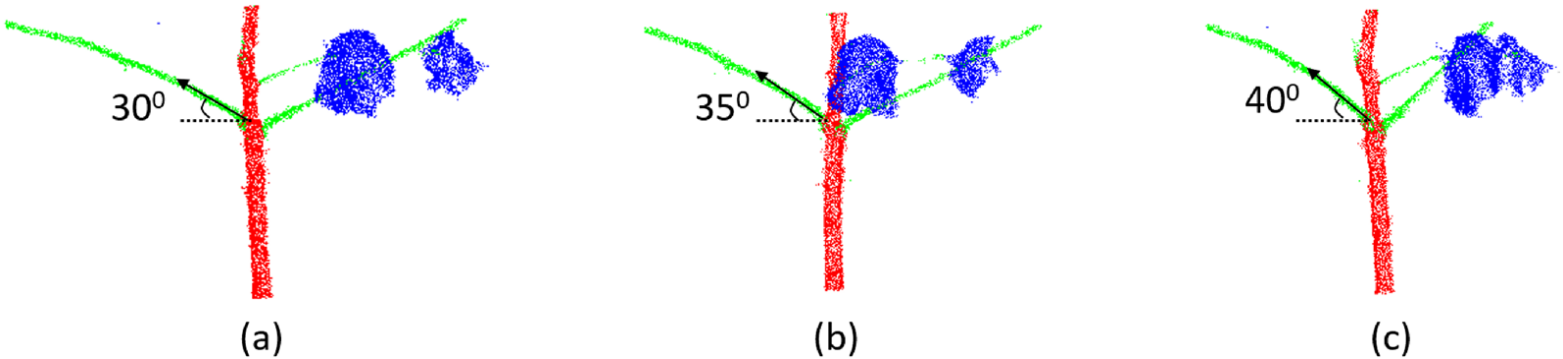
Branch inclination angle estimation from 2D views. **a** Front facing view of the plant. **b** Branch lodging away from the camera. **c** Branch lodging towards the camera

Most previous studies used 2D images to estimate boll counts [41]. As 2D images only capture a single view at a time, the hidden and highly occluded bolls cannot be detected. In contrast, the 3D point cloud sample of the cotton plant in our study captures views from all angles and has less occlusion and bolls can be captured from all angles. While 2D-image-based boll detection using fully and weekly supervised segmentation shows a strong correlation (R^2^ < 0.91) on the dataset studied by researchers, the 3D imaging approach significantly outperforms the 2D imaging approach qualitatively and shows a strong correlation on our dataset with the ground truth (R^2^ = 0.99). The presented method using 3D point clouds was more robust for architecture trait extraction than other studies based on 2D images for fruit and leaf counting [16, 17, 53]. The presented approach is also robust for cases where the branch is oriented towards or away from the camera while 2D image-based studies for automated stem angle determination are more error-prone in this case [15]. In cotton plants, this is illustrated by estimating branch inclination angles from different 2D views of the plant. 2D images of point clouds from different views including the front-facing view were captured. The estimated value of the branch inclination angle varied across different views of the same plant as illustrated in Fig. 11. The branch inclination angle estimated from the front view was lower than the angle estimated from the views where the branch lodges towards or away from the cameras. In comparison, our method based on 3D point clouds estimated the same values for the inclination angles of a branch for any view.

**Fig. 12:**
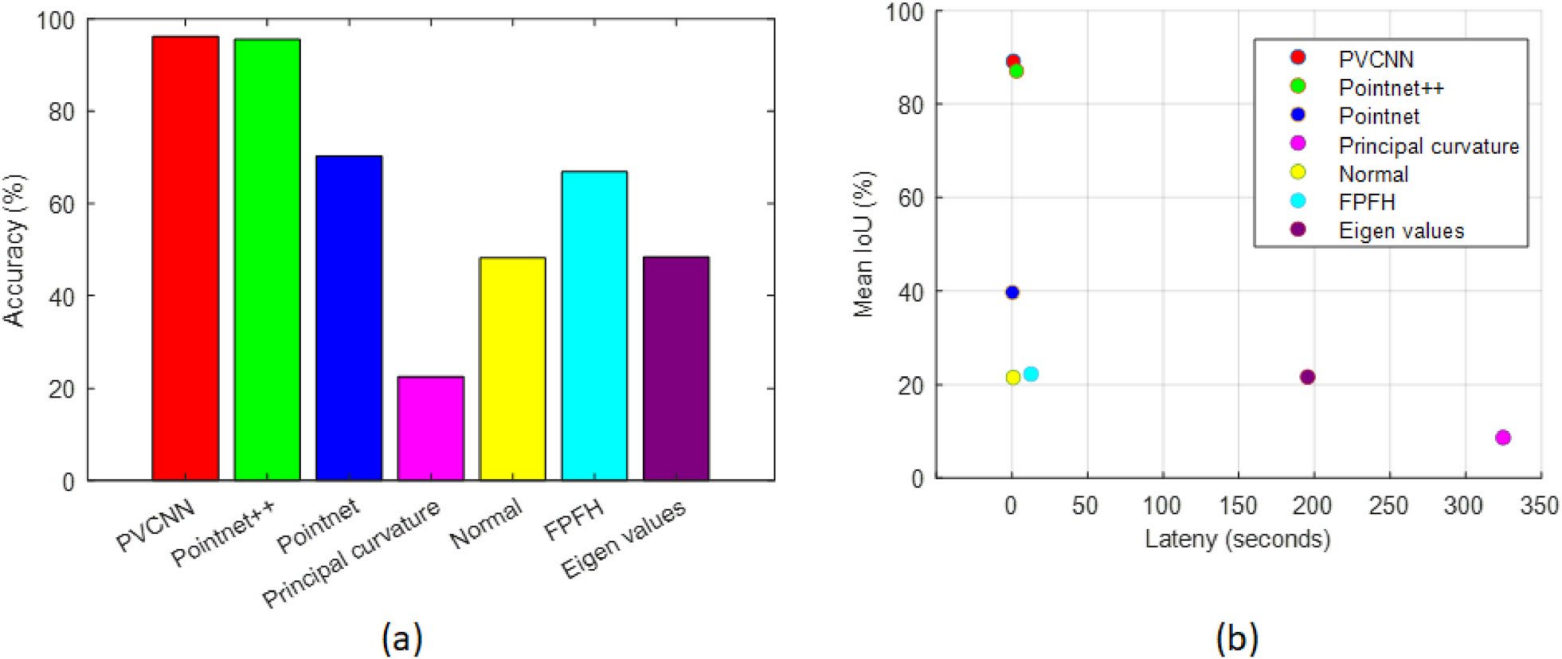
Comparison of hand-crafted features and the deep learning-based features for segmentation of main stems, branches and cotton bolls. **a** Segmentation accuracy **b** Time consumption (in seconds) vs mean IoU (%)

In comparison to other 3D point cloud-based approaches for main stem height estimation utilizing point coordinates and local surface features [29, 54] in cotton, corn, wheat, and tomato, our approach demonstrates a higher correlation between the predicted value and the ground truth. Our approach also estimates height in the curved main stem cases in contrast to [36] when a cylinder is approximated as the main stem of a barley plant and cases with tilted and curved main stems are not accounted for, resulting in less accurate main stem height estimations. Our approach outperformed the 3D approach [26] based on voxel cloud connectivity segmentation (VCCS) and color-based region segmentation (CRGS) for boll number estimation. The approach based on VCCS and CRGS utilized intensity and local surface features compared with advanced features utilized in our study. We employed a deep learning approach which is used to automatically extract features from the data that are useful for the segmentation and classification task. Other studies [36–38, 45, 55, 56] segmented grapevines, wheat, barley, sorghum, tomatoes and rose plants using handcrafted features such as eigenvalues of the local covariance matrix, fast point feature histogram (FPFH), and principal curvature. In the cotton plants dataset, the performance of handcrafted features on segmentation of the main stems, branches and cotton bolls is evaluated to compare with features extracted from 3D deep learning. The eigenvalues of the covariance matrix, FPFH, principal curvature and normal features are estimated on a local region with a radius of 1 cm. A softmax classifier is trained and performance is evaluated for each handcrafted feature. Among the handcrafted features, FPFH achieved the highest segmentation accuracy (Fig. 12a). The handcrafted features achieve less accurate results than the deep learning approach. Analysis of mean IoU and latency indicate that all deep learning-based methods excelled in mean IoU (Fig. 12b). Moreover, all handcrafted features with the exception of normal features showed substantially higher latency than PVCNN, Pointnet++ and Pointnet. In our cotton plant dataset, both the main stem and branches are tube-like shapes; therefore, the local surface features in Fig. 12 showed lower performance whereas the PVCNN utilized the hidden features in the data to classify the points more accurately.

**Fig. 13:**
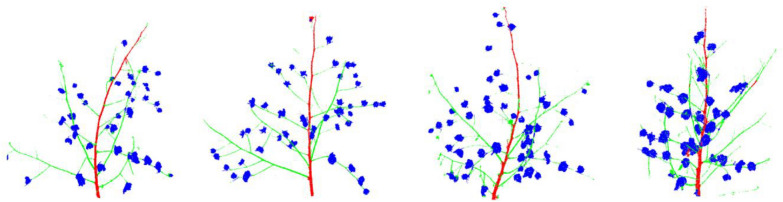
Samples of inference results on unseen and unlabeled data

To verify the claimed advantages of segmentation method, we scanned 10 more plants in indoor environment and applied preprocessing including cropping, denoising, normalization, and down-sampling. We used the trained point-voxel convolutional neural network to perform inference on this unseen and unlabeled dataset. The inference results showed that most regions were correctly segmented with the exception of a few mispredictions as represented in a sample in Fig. 13.

**Fig. 14:**
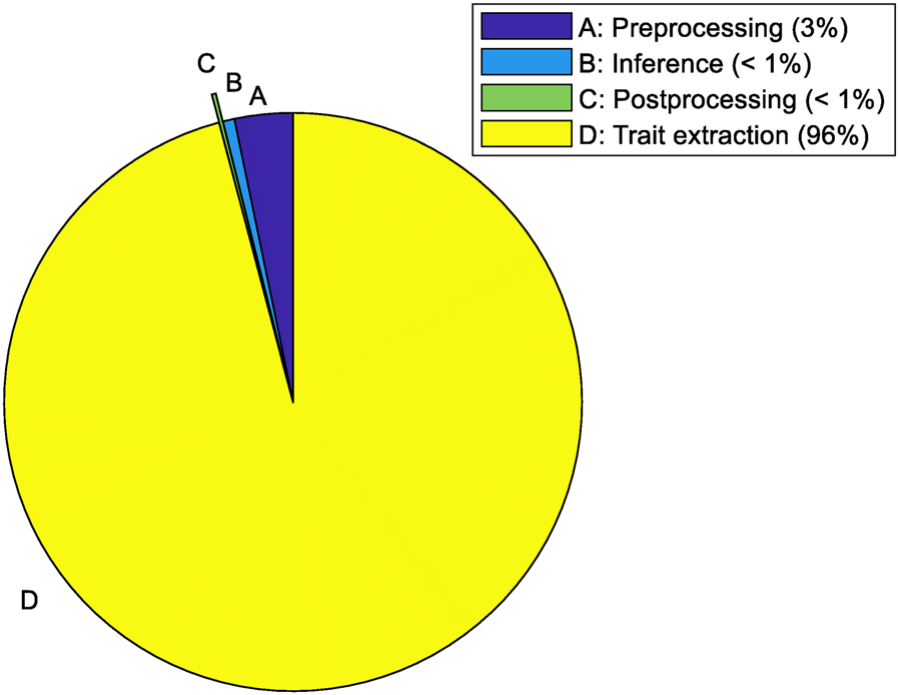
Inference time consumption of each stage for PVCNN (in seconds and the percentage to the total time consumption)

To evaluate the practicality of the presented method, we assessed the time consumed in each stage for obtaining estimates of the architectural traits. In this study, the denoising step was performed manually using CloudCompare software. For the purpose of measuring the time consumption in denoising, the statistical outlier removal of points was automated. The time consumption was recorded for the preprocessing stage which included denoising, normalizing, and down-sampling steps. Based on the results, the trait extraction stage accounted for more than 90% of the time while the preprocessing, inference and post processing stage took less than 5% of the total time (Fig. 14). The future plan is to optimize the architectural trait extraction stage to reduce its time consumption and combine and automate all steps in the processing pipeline.

In this method, part segmentation and trait extraction were performed on the single plant point clouds. To achieve our goal of cotton plant architecture characterization and part segmentation, we used ground sensing given its ability to capture finer details at the lower part of a plant. In outdoor data collection, ground sensing can easily assist in the estimation of plant-level traits including stem diameter, branch angle, branch height, number of nodes, number of branches and others. Given the flight altitude and limited resolution, the aerial imagery approach would not be feasible to capture the same level of details as our terrestrial LiDAR to characterize cotton plant architectural traits. Ground sensing may require more manual handling to fully capture the data for the entire field while the UAV can be handled remotely for capturing field-level data. Although the annotation tool developed in this study uses pointwise labeling for segmentation in a single plant, the bounding box labeling utility can be used to annotate each plant in plot-level data. Hence, this segmentation method could be modified and extended to plot-level data captured using airborne LiDAR scanners and mobile platforms. Therefore, the estimation of plot-level traits such as the number of cotton bolls per plot is planned to be investigated in future research.

## Conclusions

This study applied three 3D deep learning models to segment the main stem, branches, and bolls of cotton plants and extract architectural traits. This is particularly important in cotton, which has a more complex growth habit than most major row crop species. The custom-made PlantCloud annotation tool demonstrated more functionality with the lowest memory consumption. 3D deep learning automatically obtained useful features based on the labeled dataset which avoided the need to manually select handcrafted features. Using both point and voxel representation of 3D data, the optimal performance in terms of segmentation accuracy and efficient inference time was achieved through PVCNN among the three models. The architectural traits derived from the post-processed segmentation results showed a strong correlation with the ground truth. Overall, the plant part segmentation and architectural trait extraction results are promising and could be used for automated plant phenotyping and physiological studies.

## Materials and methods

**Fig. 15:**
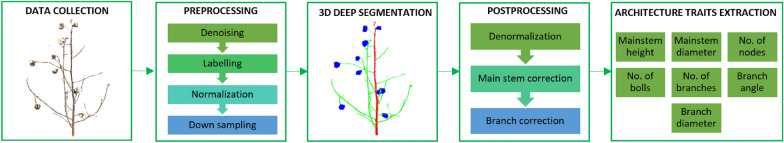
Segmentation and trait extraction workflow. Five phases including data collection, preprocessing, deep segmentation, post processing and trait extraction are carried out sequentially

There were five major components in the data processing pipeline for plant part segmentation and trait extraction (Fig. 15. First, 3D data collection was performed and the input point clouds were passed through the preprocessing stage. After preprocessing, 3D deep learning was applied for segmenting the plant parts. The predicted segments were then passed through the post processing stage. The results obtained from the post processing were used in extraction of seven architectural traits. The following sections introduce these procedures in detail.

### Data collection

**Fig. 16:**
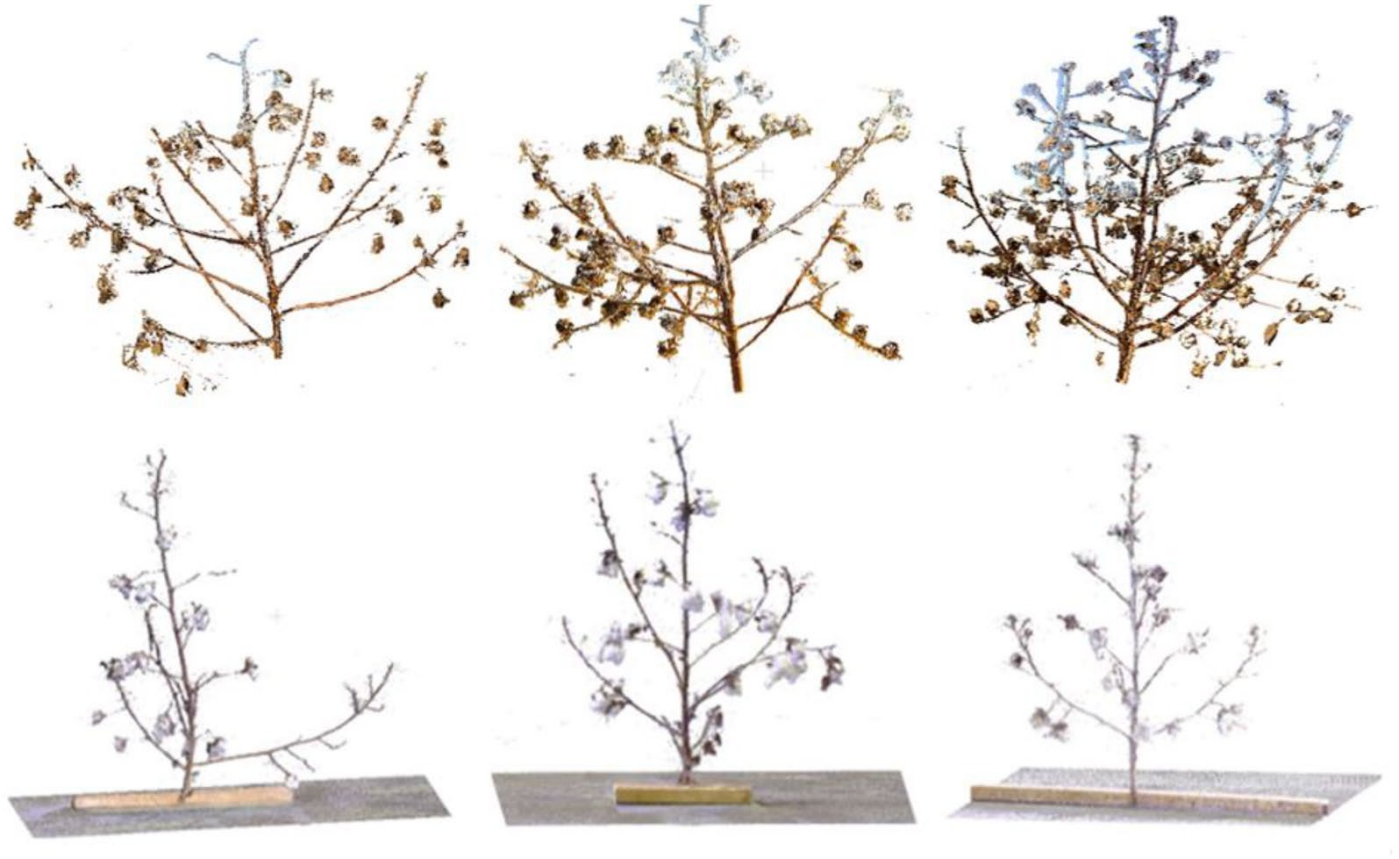
Examples of collected cotton plant point clouds. The top row represents plant data collected in-situ in an outdoor field setting in session 1. The bottom row represents data collected in an indoor setting in session 2 and 3

The raw data comprising 3D point clouds of the cotton plant were collected in three sessions at the Plant Research Farm of the University of Georgia, Athens GA, USA. The first session was held in December 2018 where the data were collected in an outdoor field setting and the plants were scanned from single-plant plots. The next two sessions were held in December 2020 and February 2021 for plants from regular plots (10 – 15 plants per plot). In these sessions, the plants were cut from their base and brought to an indoor setting for data collection. Each plant was fixed to a wooden base to arrange in a standing position. FARO Lidar Scanner was used for 3D data collection in all sessions. The measurement unit in the FARO LiDAR device was set to ‘meters’ and accordingly the distance between any two points in the scans was set in meters. Spherical targets were used in data collection for registering scans from different angles. The recorded LiDAR scans were registered using SCENE software [57] and point clouds for individual plants were obtained. An illustration of point clouds for a sample of plants shows that plants from session 1 are wider in structure compared to plants from the other two sessions (Fig. 16). Moreover, some plant parts in the point cloud data are brighter than others because of differences in illumination. The overall dataset covers point clouds of 30 individual cotton plants. There are on average more than 450,000 points per point cloud. More than 70% of the points in all datasets belong to the cotton boll class, whereas the main stem and branches combined cover less than 30% of the total points. Each point consists of x, y, z, R,G, and B as a six-vector.

### 3D annotation software

To prepare the annotated dataset of plant point clouds, we developed the 3D annotation software PlantCloud. The main purpose of the annotation software is to allow pointwise labeling and bounding box annotation of plant parts.

We developed the annotation software in C++ and OpenGL to achieve efficient annotation, manipulation and rendering of high-resolution plant point clouds. Using C++ and OpenGL, the plant annotation software directly interacts with the system’s graphics card without any intermediate applications (such as web browser or ROS). The overall development of the software was divided into two parts. The first part consisted of developing a module to render the point clouds from different angles and positions. The second part included the user interface design and development of a module to allow pointwise labeling and bounding box annotation.

**Fig. 17:**
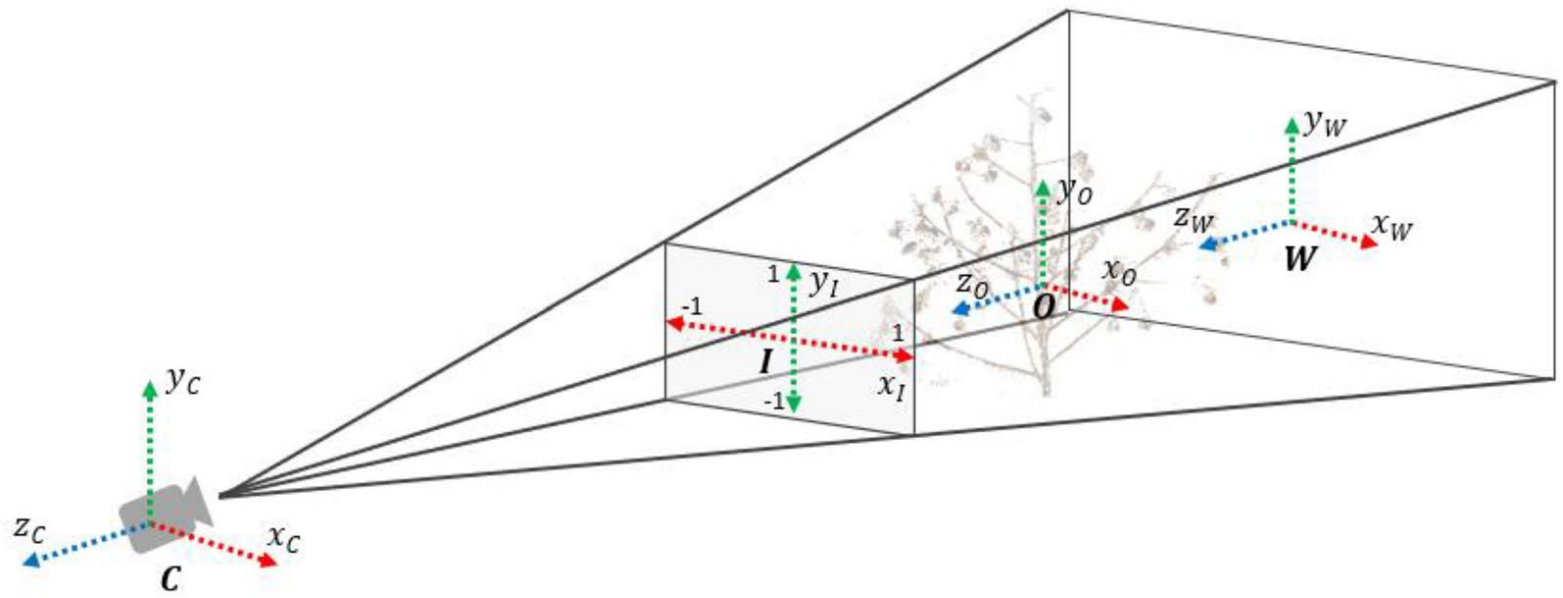
Coordinate system representation for rendering plant point cloud on screen. $O, W, C, I$ represent object, world, camera, and image coordinate systems, respectively

To render the plant point clouds in each frame, a series of transformations were applied on input point cloud to transform through multiple coordinate systems (Fig. 17) and to finally achieve coordinates in the screen space. The input point cloud was initially in object coordinate system which was relative to local origin. Using the ‘model matrix’, these local coordinates were transformed through rotation and translation to world space coordinates which were relative to global origin in the world. The world coordinates were transformed to camera space coordinates using the ‘view matrix’ in such a way that each coordinate was as seen from the camera. The camera space coordinates were then projected to 2D space as image plane coordinates using the ‘projection matrix’. These coordinates were normalized in the -1 to 1 range. Lastly, the image plane coordinates were transformed to screen coordinates using the ‘viewport transformation’ which transformed the normalized coordinates to coordinate range defined by the device screen (for example, the coordinate range is (0,0) to (1920,1080) for a 1920 × 1080 dimension screen).

**Fig. 18:**
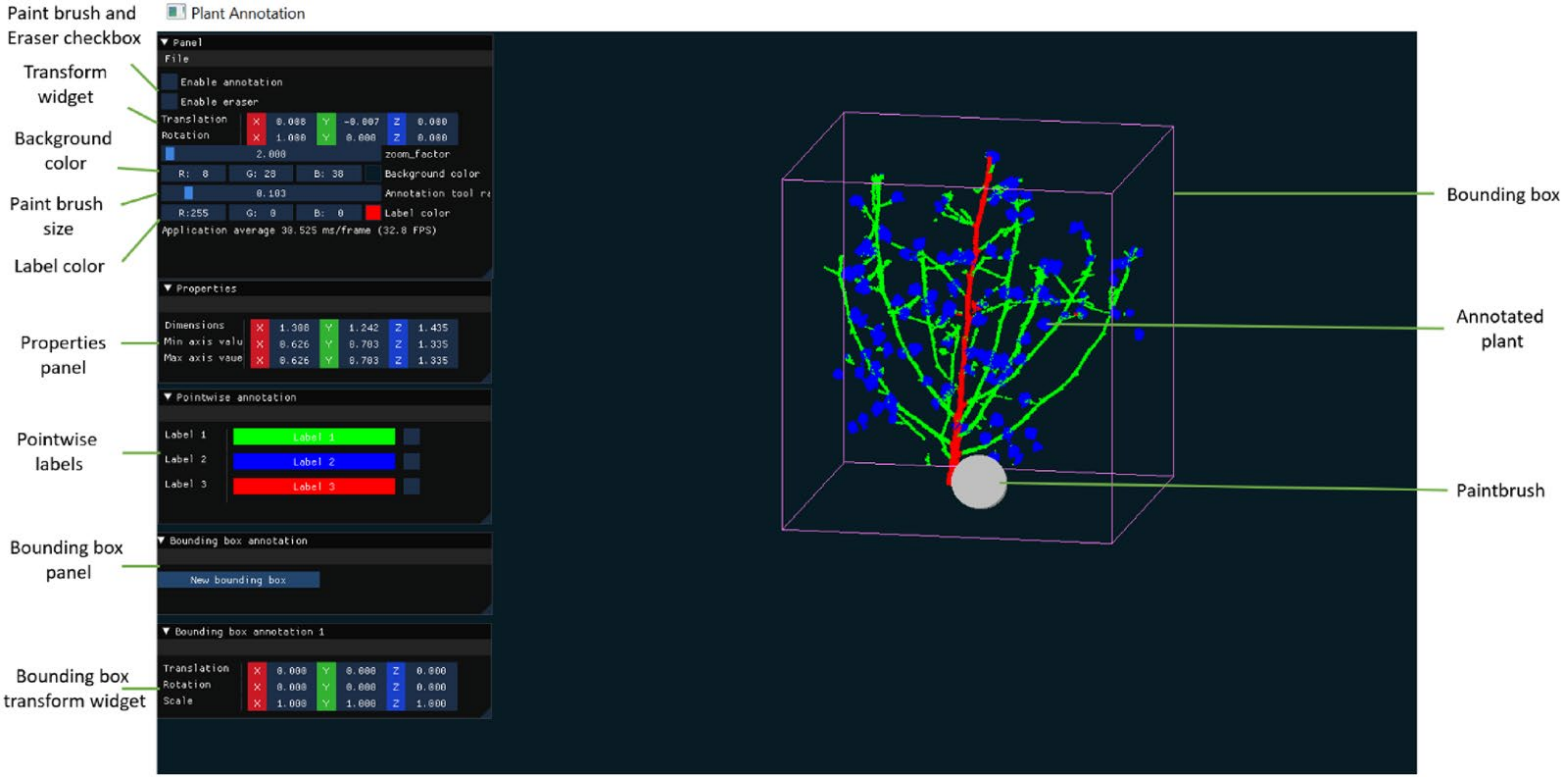
User interface of PlantCloud software for plant 3D point cloud annotation

To design the user interface with cross platform support, the open source library ‘ImGui’ was leveraged in C++. The overall interface (as shown in Fig. 18) was designed to enable the user to perform both pointwise labeling and bounding box annotations. The user interface enables users to adjust point cloud position through rotation and translation using mouse drag and drop as well as the transform widget. The software enables pointwise labeling through the paint brush, the label color selected by users and the labels panel. For bounding box annotation, the software allows for the addition of a new box with associated transform widget in the bounding box panel, for adjusting the box size and position. Along with annotation support, the software displays the point cloud dimensions in the properties panel. After a plant is fully annotated, the software saves the annotated point cloud at a desired location using a file dialog.

### Data preprocessing

**Fig. 19:**
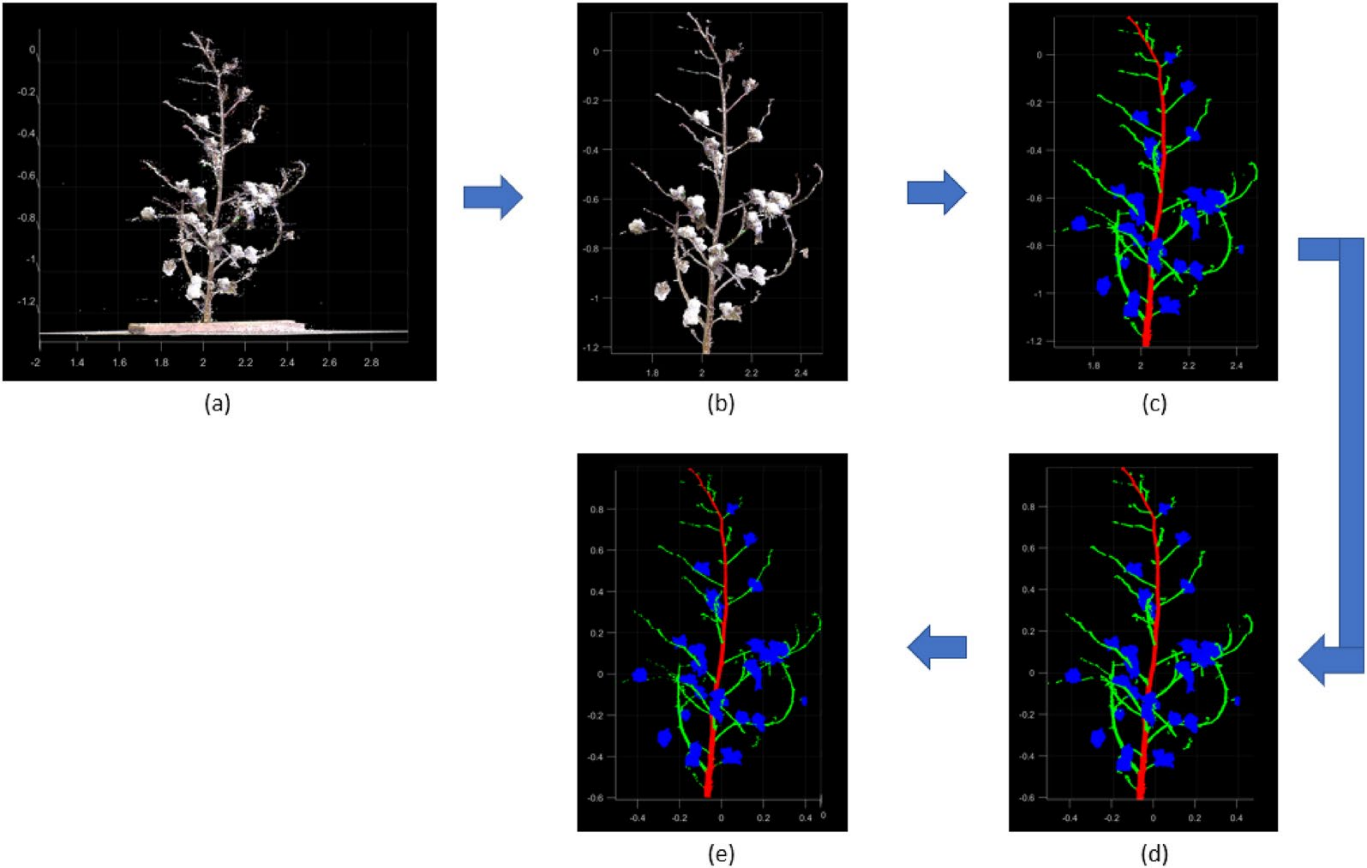
Data preprocessing steps on a sample input point cloud. **a** Input point cloud. **b** Denoised point cloud. **c** Labeled point cloud. **d** Normalized point cloud. **e** Down sampled point cloud

Data preprocessing was applied on input point clouds for denoising, annotation, normalization, and down sampling before segmenting the plant parts (Fig. 19).

*Denoising:* The input point clouds acquired from the registered LiDAR scans contain noise. For denoising, the statistical outlier removal method in CloudCompare software [58] was used. Using this method, the average distance of each point from 6 nearest neighbors was estimated and points exceeding a distance of 1 standard deviation were removed to obtain a denoised point cloud (Fig. 19b).

*Annotation:* On the input point clouds, point-wise annotation of cotton plants was performed to prepare a labeled dataset of cotton plant part segmentation. Using annotation software, the main stem, branches, and cotton bolls of the dataset were manually labeled in red, green, and blue color respectively as illustrated in Fig. 19c.

*Normalization:* As the point clouds can be at varying scales, they were normalized to unit sphere so that all point clouds were at a common scale (Fig. 19d). For normalization, first the point cloud center needs to be shifted to zero. To achieve this, the average of all point coordinates, $\widehat{p}=\left(\widehat{x},\widehat{y},\widehat{z}\right)$ was estimated using Eq. 1. It was subtracted from each point ${p}_{i}=\left({x}_{i},{y}_{i},{z}_{i}\right)$ in the sample to achieve a mean of 0. Afterwards all point coordinates were divided by the maximum norm of all points in the sample to result in normalized point coordinates (Eq. 2). The radius of the resulting point cloud was 1.

1 $$\widehat{p}=\left(\widehat{x},\widehat{y},\widehat{z}\right) = \frac{1}{n}\sum_{1=1}^{n}\left[{x}_{i},{y}_{i},{z}_{i}\right]$$

where n represents the total number of points in the sample.

2 $${p}_{i}\mathrm{^{\prime}}=\frac{{p}_{i}-\widehat{p}}{\underset{{p}_{k}\in P}{\mathrm{max}}\left(norm({p}_{k})\right)}$$

where norm is calculated as the Euclidean distance from the origin (0,0,0).

3 $$\mathit{norm}\left({p}_{i}\right) =\sqrt{{x}^{2}+{y}^{2}+{z}^{2}}$$

In inference, each point cloud was denormalized back to its original state so that architectural traits such as stem height, stem, and branch diameter can be extracted in meters.

*Down sampling:* The point cloud for each cotton plant have more than 400,000 points on average. Because of hardware limitations, applying 3D deep learning on the entire point cloud is unfeasible. When using a Tesla V100 GPU card, the method exceeds memory limitations after the number of points exceeds 110,000. To leverage maximum information, each point cloud was randomly down-sampled to 100,000 points which did not exceed memory limitations (Fig. 19e).

### 3D deep learning approaches

This section first gives an overview of Pointnet and Pointnet++ networks and then describes the Point Voxel Convolutional Neural Network (PVCNN) for achieving better performance.

#### Pointnet

**Fig. 20:**
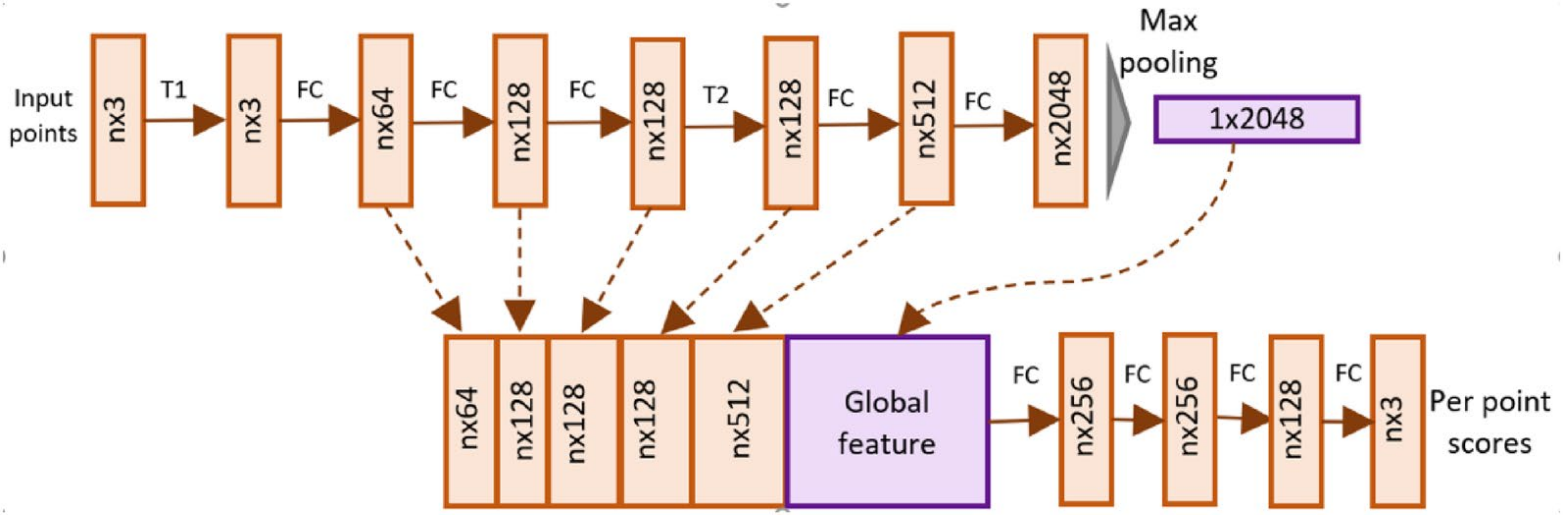
Pointnet architecture. The network takes n points as input, applies feature extraction. It then aggregates the point features by max pooling and concatenates the features to output scores per point. FC is fully connected layer operating on each point. T1 and T2 are transformation networks for input points and features

Pointnet is among the pioneering 3D deep learning architectures that directly consumes point clouds for performing 3D tasks of segmentation and classification. The network is invariant to the order of points in the input point cloud and works by extracting features from individual points. It then aggregates the global information by applying pointwise max pooling operation on extracted features. The aggregated global feature is concatenated with individual point features. Afterwards, a multi-layer perceptron classifier is trained to output scores per point for each part. In the network architecture adopted in our study, the skip connections are used to combine individual point features from all previous layers (Fig. 20). The combined feature for each point is concatenated with the global feature and fed to the multilayer perceptron network for classification into the main stem, branch, and cotton boll classes.

#### Pointnet++ 

The Pointnet network operates on each point individually and aggregates the global information. As a result, it does not consider the local neighborhood information of a point thus limiting its ability to recognize fine-grained patterns. To address this issue, Pointnet++ architecture was adopted since it considers the information from the surrounding neighborhood of a point within a certain radius. Pointnet++ architecture achieves this purpose by using the set abstraction and feature propagation layers.

The set abstraction layer involves sampling, grouping and aggregation step. The sampling step selects k number of points using the farthest point sampling method. The selected points define the centroid of local regions. The grouping step is then performed to gather the neighboring points around each centroid within a certain radius. Finally, the set abstraction layer aggregates the neighborhood of each centroid using MLP layers. The set abstraction layer can form groups of a point at single or multiple radius levels. The information of groups from each radius level is aggregated and concatenated to form a final aggregated feature vector of centroids.

**Fig. 21:**
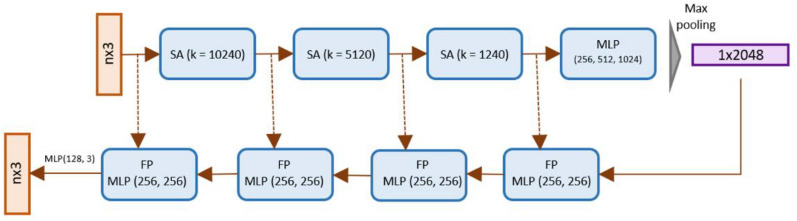
Pointnet++ architecture. Three set abstraction (SA) layers are applied and followed with MLP layer. The resulting features are aggregated using Max pooling and interpolated using four Feature propagation (FP) layers. The FP layers are followed by MLP layers and the final MLP layers are used to output scores per point

The feature propagation layer interpolates the estimated features for all points. As the Pointnet++ applies a series of set abstraction on a sample of points, features for the remaining points in the original point cloud need to be interpolated to achieve pointwise scores. For this purpose, feature propagation layers are applied corresponding to each set abstraction layer. The sampled points of each set abstraction layer are used to interpolate features for points in the input set of that layer. In addition to performing interpolation, the feature propagation layer also uses skip connections with the corresponding set abstraction and MLP network for richer information as shown in the architecture of Pointnet++ adopted in our study (Fig. 21). In our customized network architecture, we employed 3 set abstraction layers with the sample values of 10,240, 5120, and 1240. Further we leveraged multiscale grouping. In this way local features of a point are extracted at different radius levels. The features extracted at all radius levels are concatenated for a point.

#### Point voxel convolutional neural network (PVCNN)

**Fig. 22:**
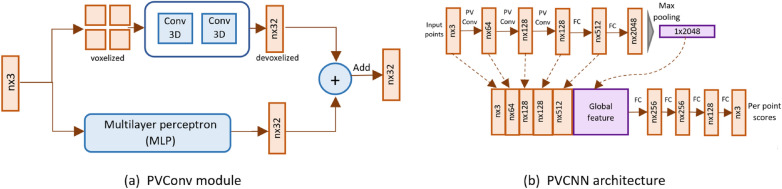
Point Voxel Convolutional Neural Network. **a** The PVConv layer applies 3D Convolution on voxelized input. It devoxelises the aggregated features back to map the points. The point-based and voxel-based features are fused to form the output. **b** The PVCNN network takes n points as input and applies PVConv and FC layers. It then aggregates the point features by max pooling and concatenates the features to output scores per point

The Pointnet and Pointnet++ architectures utilize the point-based representation of 3D data. Liu, Tang [47] showed performance enhancement by using both point- and voxel-based representation through Point Voxel Convolution Neural Network (PVCNN). The PVConv module is employed to combine both point- and voxel-based representations. The PVConv module comprises two branches for computing features from both voxel- and point-based representation of the input point cloud (Fig. 22). The features from the two branches are fused using an addition operation to form the output for PVConv module. The PVCNN architecture adopted in our experiment is formed by replacing initial fully connected layers of Pointnet with PVConv module (Fig. 22b). In our customized network architecture, we use four consecutive PVConv layers followed by MLP layers. The voxel resolution is set to 128 and 100 for the first and next two PVConv layers, respectively.

### Experiment settings

In the experiment phase, the dataset was divided into training and testing set with the ratio of 70% to 30%. The RGB information was excluded due to differences in illumination and only point coordinates were considered in segmentation. In the training phase, the data augmentation was performed on the point clouds in each iteration. The point clouds in the training set were randomly rotated along x and y axis with the probability of 0.5 and 0.3 respectively. Afterwards, all networks were trained for 300 epochs with the initial learning rate of 0.001. A Tesla V100 GPU card was used in the entire process of training and testing.

#### Evaluation metrics

The overall segmentation performance was evaluated through mean Intersection over Union (mIOU) and accuracy. For mIoU estimation, the IoU for all classes per point cloud was first averaged. These averaged IoUs for each point cloud were used to calculate the final mIoU by taking their mean over all point clouds. The accuracy was calculated as the percentage of correctly classified points from the total number of points. The efficiency of the method was evaluated in terms of average inference time per point cloud.

The segmentation performance of each class was evaluated in terms of Intersection over Union (IoU), Recall and Precision. The Precision (Eq. 4) of a class is defined as the proportion of correct detections from all detections of that class, while Recall (Eq. 5) as the proportion of detected points from all the points belonging to a class. The IoU (Eq. 6) of a class is evaluated as the ratio of the common region to the overall region of ground truth and predicted segments belonging to that class.

4 $$Precision = \frac{{TP}_{k}}{{TP}_{k} + {FP}_{k}}$$

5 $$Recall = \frac{{TP}_{k}}{{TP}_{k} + {FN}_{k}}$$

6 $$IOU = \frac{{TP}_{k}}{{TP}_{k} + {FP}_{k} + {FN}_{k}}$$

where ${TP}_{k}$, ${FP}_{k}$, ${FN}_{k}$ represent true positives, false positives, and false negatives for a class k respectively.

### Postprocessing

On the predicted segments of plants, further post processing was applied before proceeding for architectural trait extraction. Firstly, the point clouds were denormalized back to the original state so that distance between any two points can be estimated in meters. Next, it was observed in segmentation results (discussed in “Segmentation results” Section) that small parts of the main stem were incorrectly predicted as branch points whereas small parts of branches were mis-segmented as the main stem. Main stem and branch corrections were applied to address this issue. In all point clouds, the bottom-most points of the main stem were predicted correctly. Therefore, to apply main stem correction, the bottom-most 1 cm slice of the main stem was selected and its bounds along x and y axes were noted. Afterwards, the 1 cm slices along the z axis were iteratively selected up to the slice with highest main stem prediction to apply the branch and main stem correction. In each slice selection, the non-main stem points lying within the noted bounds (i.e. the maximum and minimum value along x and y axes of main stem predictions in the previous slice) after keeping a margin of 1 cm, were corrected as main stems while those lying outside were corrected as branches. From the main stem points in the selected slice, the bounds along the x and y axes were noted to be used in the next iteration for main stem and branch correction. This procedure is outlined in Algorithm 1. This method filled the parts in the middle of the main stem containing incorrect branch predictions. Because we checked the bounds in every iteration, we have considered the assumption that the main stem cannot change growing direction abruptly. This process was repeated for two rounds with slightly different margins.
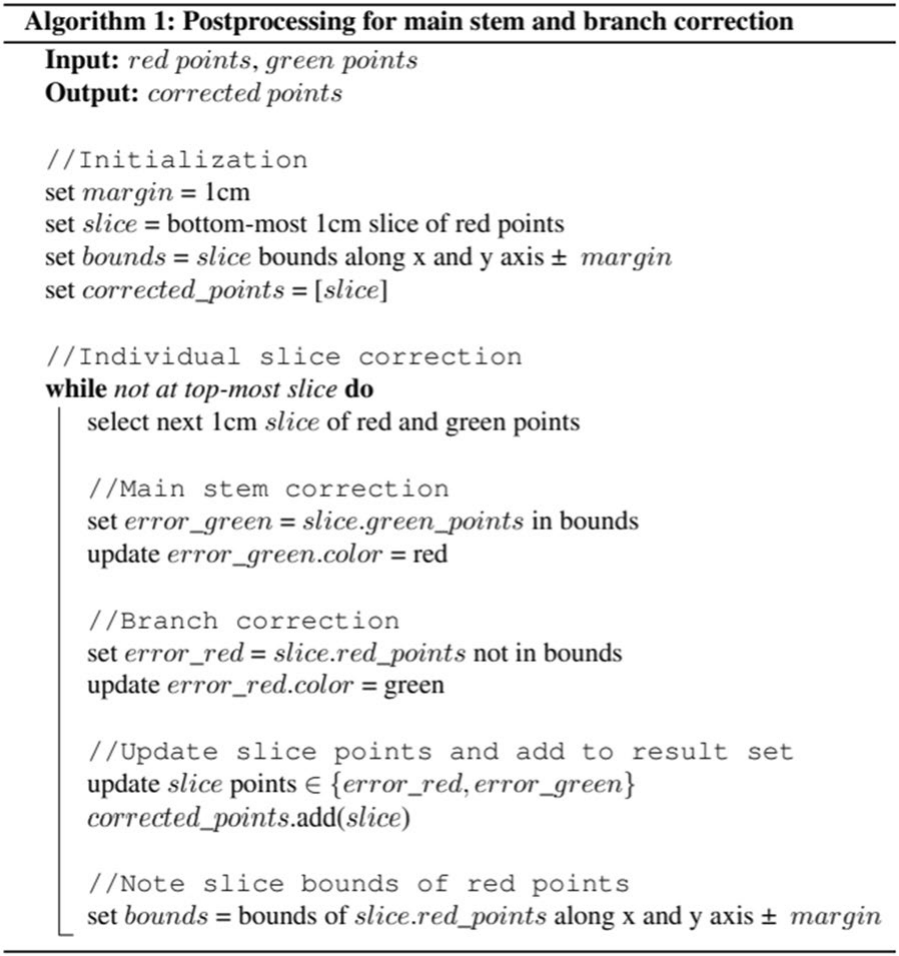


### Architectural traits extraction

Seven architectural traits of cotton plants were extracted from the segmented parts and the traits include main stem diameter, main stem height, number of nodes, number of branches, number of bolls, branch inclination angle and branch diameter. The predicted traits were compared with the ground truth estimated from manual measurements. For node and branch detection, crop physiologists typically do not consider the top part of the plant above the uppermost harvestable boll. This is because cotton is an indeterminate plant, in which the regrowth of new vegetative tissue begins after the crop has reached physiological maturity. This new growth does not contribute appreciably to yield [59]. Therefore, the branches and nodes in the part of the plant above the uppermost boll were not detected.

For main stem diameter estimation, we selected points belonging to the main stem in the lowest 1 cm region. The selected points were projected on the xy plane and a circle was fit on the projected points using the Pratt method [60]. The radius and diameter were estimated from the fitted circle. The main stem height was defined as the vertical height of a segmented main stem which may be straight, curved, or tilted. It should be noted that the height of the main stem cannot be estimated as the height of the entire plant because in some cases the branches of the plant exceeded the main stem along the z-axis. Therefore, the main stem height was calculated by taking a difference between the highest and the lowest points belonging to the main stem class.

**Fig. 23:**
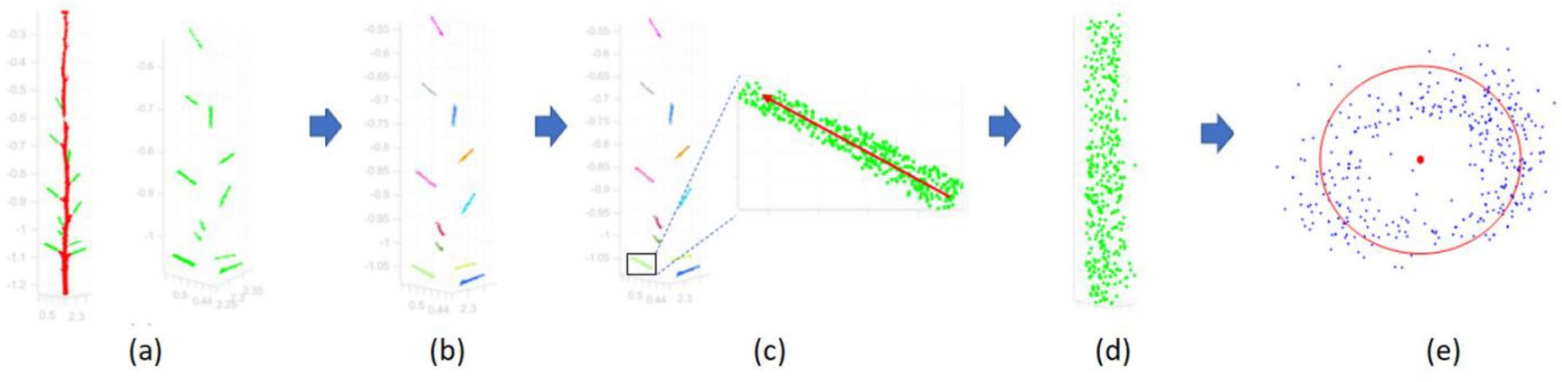
Branch traits extraction. **a** Segmented main stem and branch points within 6 cm from main stem. **b** Clustering of selected branch points. Each color represents a detected branch. **c** Principal component analysis applied on selected branch. Vector in red represents the principal component in the direction of branch **d** Vertical alignment of selected branch **e** Projection of points on xy plane and circle fitting

The branches were detected by applying DBSCAN (Density-Based Spatial Clustering of Applications with Noise) Clustering. In the plant point clouds, there were a few small branches with less than 100 points. These small branches were filtered by clustering all branch and cotton boll predictions together and removing the smaller clusters. During clustering, the ‘minPoints’ parameter was set as 100. Moreover, some gaps were observed in the branches in a few cases because of missing data therefore the ‘eps’ parameter in DBSCAN was set as 2 cm. This parameter ensured that branches with gaps less than 2 cm were considered as single cluster. After applying DBSCAN, the clusters with less than 100 points were removed and the branch predictions in retained clusters were considered for branch detection. For this purpose, the branch predictions within 2 cm radius of main stem predictions were selected. This radius was increased to 6 cm for branch inclination angle estimation, which allowed enough branch part to be selected for more precise estimation of branch inclination angle (Fig. 23a). For detecting each individual branch, clustering was applied on selected branch points with ‘eps’ value of 2 cm. The initial clustering (applied for filtering small branches) was not utilized here to detect each branch as it was applied on all branch points and multiple branches were identified as a single cluster due to small distance between them. However, branches were far apart from each other at their point of attachment of the main stem (within 2 cm distance from the main stem) and there were rare cases of nearby branches at the attachment point. As a result, the branch points lying within 2 cm from the main stem were selected and DBSCAN was applied (Fig. 23b). The resulting clusters were considered as detected branches. The number of clusters was considered as the number of branches and the points in each cluster within 1 cm from the main stem were averaged and considered as a detected branch location. For each detected branch, branch inclination angle and branch diameter were estimated. The principal component analysis (PCA) was applied on a cluster for the selected branch to estimate the branch inclination angle, which allowed us to find the most dominant component in a branch as shown in Fig. 23c. Branch inclination angle was then computed as the angle of the dominant principal component from its projection on the xy plane. For the branch diameter estimation, the selected branch cluster was rotated to align vertically. This was achieved by first rotating the dominant principal component to align it along the z-axis. The rotation matrix for this purpose was computed as follows. Let $A$ be the dominant principal component along the branch and $B$ be the z-axis, then rotation matrix to rotate $A$ to $B$ on a plane with a normal $A\times B$ is given as,

7 $$G = \left[\begin{array}{ccc}\mathrm{cos}\theta & -\mathrm{sin}\theta & 0\\ \mathrm{sin}\theta & \mathrm{cos}\theta & 0\\ 0& 0& 1\end{array}\right]$$

where $\theta$ is the angle between $A$ and $B$.

Using the rotation matrix in Eq. (7), the transformation was applied on selected branch points so that the branch is vertically aligned along the z-axis (Fig. 23d). From the vertically aligned branch part, the diameter was computed using the procedure similar to main stem diameter estimation method. The bottom-most 1 cm slice of branch was selected and projected on the xy plane. The fitted circle was used in diameter estimation (Fig. 23e).

The clusters for detected branches were also used in detecting the node positions in the main stem. For the node detection, minimum value along the z-axis for each cluster was retrieved. Afterwards, the clusters were sorted in the ascending order of their retrieved minimum values. Among the list of sorted clusters, if a clusters minimum was within 1 cm of the previous cluster’s minimum, both clusters were considered to belong to a single node otherwise the clusters were connected to two different nodes. For computing the node’s location, the branch clusters belonging to that node were considered. Afterwards the main stem slice corresponding to the associated branch clusters were selected. The points in the selected main stem slice were averaged to represent the detected node location.

For estimating the number of cotton bolls, DBSCAN was used to cluster the cotton boll segments. It was observed that there were many nearby bolls with less than 1 cm of distance between them. Hence, the ‘eps’ parameter value was set as 5 mm. The ‘minPoints’ parameter value was set as 100. The small clusters with less than 3 cm of height were removed to retain the mature bolls. The number of clusters represent the number of cotton bolls. However, in a few cases, some cotton bolls were connected with less than 5 mm distance between each other. These bolls were clustered as a single boll, which resulted in the number of clusters to be less than the number of cotton bolls. The problem of frequently occurring connected cotton bolls was previously addressed in multi plant point cloud [26]. To address the problem of a few cases of connected cotton bolls in single plant point clouds, the size of each cluster was estimated in terms of the number of points. From all the clusters, an outlier cluster was determined if it has a size more than 1.5 interquartile ranges above the upper quartile. As each cluster in most cases represents a single cotton boll, the approximate number of cotton bolls in the outlier cluster was estimated by dividing the size of that cluster by the average size of all clusters and rounding off in the end.

## Data Availability

The datasets used and/or analyzed during the current study are available from the corresponding author on reasonable request.
